# Causes of short birth interval (*kunika*) in Bauchi State, Nigeria: systematizing local knowledge with fuzzy cognitive mapping

**DOI:** 10.1186/s12978-021-01066-2

**Published:** 2021-04-06

**Authors:** Ivan Sarmiento, Umaira Ansari, Khalid Omer, Yagana Gidado, Muhammad Chadi Baba, Adamu Ibrahim Gamawa, Neil Andersson, Anne Cockcroft

**Affiliations:** 1 CIET-PRAM, Department of Family Medicine, McGill University, 5858 Chemin de la Côte-des-Neiges, 3rd floor, Montreal, QC H3S 1Z1 Canada; 2 Centro de Investigación de Enfermedades Tropicales (CIET), Universidad Autónoma de Guerrero, Acapulco, Mexico; 3 Federation of Muslim Women Association of Nigeria (FOMWAN), Bauchi State, Nigeria; 4 Bauchi State Primary Health Care Development Agency, Bauchi State, Nigeria

**Keywords:** Birth spacing, Fuzzy cognitive mapping, Deliberative dialogue, Reproductive health, Contraception, Participatory methods

## Abstract

**Background:**

Short birth intervals, defined by the World Health Organization as less than 33 months, may damage the health and wellbeing of children, mothers, and their families. People in northern Nigeria recognise many adverse effects of short birth interval (*kunika* in the Hausa language) but it remains common. We used fuzzy cognitive mapping to systematize local knowledge of causes of *kunika* to inform the co-design of culturally safe strategies to address it.

**Methods:**

Male and female groups in twelve communities built 48 maps of causes and protective factors for *kunika*, and government officers from the Local Government Area (LGA) and State made four maps. Each map showed causes of *kunika* or no-*kunika*, with arrows showing relationships with the outcome and between causes. Participants assigned weights for the perceived strength of relationships between 5 (strongest) and 1 (weakest). We combined maps for each group: men, women, and government officers. Fuzzy transitive closure calculated the maximum influence of each factor on the outcome, taking account of all relationships in the map. To condense the maps, we grouped individual factors into broader categories and calculated the cumulative net influence of each category. We made further summarised maps and presented these to the community mapping groups to review.

**Results:**

The community maps identified frequent sex, not using modern or traditional contraception, and family dynamics (such as competition between wives) as the most influential causes of *kunika*. Women identified forced sex and men highlighted lack of awareness about contraception and fear of side effects as important causes of *kunika*. Lack of male involvement featured in women’s maps of causes and in the maps from LGA and State levels. Maps of protective factors largely mirrored those of the causes. Community groups readily appreciated and approved the summary maps resulting from the analysis.

**Conclusions:**

The maps showed how *kunika* results from a complex network of interacting factors, with culture-specific dynamics. Simply promoting contraception alone is unlikely to be enough to reduce *kunika*. Outputs from transitive closure analysis can be made accessible to ordinary stakeholders, allowing their meaningful participation in interpretation and use of the findings.

**Plain English summary:**

For people in Bauchi State, northern Nigeria, *kunika* describes a short interval between successive births, understood as becoming pregnant again before the previous child is weaned. They recognise it is bad for children, mothers and households. We worked with 12 communities in Bauchi to map their knowledge of the causes and protective factors for *kunika*. Separate groups of men and women built 48 maps, and government officers at local and state level built four maps. Each group drew two maps showing causes of *kunika* or of no-*kunika* with arrows showing the links between causes and the outcome. Participants marked the strength of each link with a number (between 5 for the strongest and 1 for the weakest). We combined maps for women, men and government officers. We grouped similar causes together into broader categories. We calculated the overall influence of each category on *kunika* or no-*kunika* and produced summary maps to communicate findings.

The maps identified the strongest causes of *kunika* as frequent sex, not using modern or traditional contraception, and family dynamics. Women indicated forced sex as an important cause, but men focused on lack of awareness about contraception and fear of side effects. The maps of protective factors mirrored those of the causes. The groups who created the maps approved the summary maps. The maps showed the complex causes of *kunika* in Bauchi. Promoting contraception is unlikely to be enough on its own to reduce *kunika*. The summary maps will help local stakeholders to co-design culturally safe ways of reducing *kunika*.

**Supplementary Information:**

The online version contains supplementary material available at 10.1186/s12978-021-01066-2.

## Non-English language abstract (Spanish)

**Antecedentes:**

Los intervalos intergenésicos cortos (menores de 33 meses, según la OMS) afectan la salud y el bienestar de la madre, el niño y la familia. Aunque los habitantes del norte de Nigeria reconocen muchos efectos adversos de un intervalo intergenésico corto (*kunika* en lengua hausa), éstos aún son frecuentes. Nosotros usamos cartografía cognitiva para sintetizar el conocimiento local sobre causas de *kunika* y guiar el codiseño de estrategias culturalmente seguras que permitan su disminución.

**Método:**

Grupos de hombres y mujeres en doce comunidades hicieron 48 mapas, mientras funcionarios del Estado de Bauchi y del Área Local de Gobierno (LGA) hicieron otros cuatro. Cada mapa mostraba causas de *kunika* o de no-*kunika* con flechas indicando la influencia entre ellas. Los participantes ponderaron la influencia entre 1 (la más débil) y 5 (la más fuerte). Nosotros combinamos los mapas por grupos de mujeres, hombres y funcionarios. Con *fuzzy transitive closure* calculamos la máxima influencia entre factores cuando todas las relaciones en el mapa son consideraras. Condensamos los mapas agrupando factores individuales en categorías y calculamos la influencia neta acumulativa para cada una. Estos mapas los sintetizamos aún más para revisarlos con sus autores.

**Resultados:**

Los mapas de los grupos comunitarios identificaron el sexo frecuente y factores relacionados, no usar anticonceptivos modernos o tradicionales y las dinámicas familiares (como el deseo de tener más hijos o la competencia entre esposas) como las causas más importantes de *kunika*. Los mapas de las mujeres identificaron el sexo forzado como causa importante y los mapas de los hombres destacaron la falta de conocimiento sobre anticoncepción y el temor a los efectos secundarios. La falta de participación masculina apareció en los mapas de las mujeres, del LGA y del Estado. Los mapas de los factores protectores reflejaron en gran medida los de las causas. Los grupos comunitarios aprobaron y apreciaron los mapas que sintetizaban el análisis.

**Conclusiones:**

Los mapas comunitarios mostraron que *kunika* es el resultado de una compleja red de factores con dinámicas culturales específicas. Es poco probable que enfocarse solo en promover anticoncepción reduzca *kunika*. Los resultados del *transitive closure* pueden comunicarse al público en general para una mayor participación en la interpretación y uso de los resultados.

## Background

A substantial body of evidence links short birth interval with child morbidity and mortality [1, 2]. One analysis suggested that avoiding birth intervals of less than 36 months could have averted 35% of deaths among children under five years old in developing countries [3]. The World Health Organization (WHO) recommends a birth to pregnancy interval of at least 24 months, equivalent to a birth interval of about 33 months [4]. A recent analysis of Demographic and Health Survey data from 77 countries suggested that child health risks of short birth interval are mostly confined to low- and middle-income countries [5]. The evidence for adverse maternal health consequences of short birth interval is mixed [6]. The causal mechanisms for maternal and child health consequences of short birth interval are not fully understood [7]. Some of the reported associations between short birth interval and adverse outcomes might be due to confounding by socio-economic factors [8].

Short birth interval is a well-recognised phenomenon in northern Nigeria; the word *kunika* in the Hausa language means getting pregnant before the last child is weaned. Northern Nigeria is predominantly Muslim, and Islamic teaching recommends breastfeeding for 24 months [9], so avoiding *kunika* aligns closely with the WHO recommendation of a birth to pregnancy interval of not less than 24 months. Islamic scholars in Nigeria discourage *kunika* because of its risks for the health of the mother and child [9]. Our recent community focus group discussions in Toro Local Government Area (LGA) in Bauchi State, Northern Nigeria, confirmed that men and women had a good understanding of the concept of *kunika* and considered it to be a bad thing, threatening the health and well-being of mothers, children, and fathers. The group participants also described how *kunika* could lead to economic hardship, conflict within families and social disapproval [10].

A recent systematic review examined the quantitative and qualitative evidence about factors related to short birth interval. For most factors, the evidence from quantitative studies was mixed; only shorter duration of breastfeeding and a female previous child were consistently associated with short birth interval [11]. Qualitative studies included in the review explored a broader range of factors including communication between couples and families, local media and social influence, and observation of other parents [11].

Men in northern Nigeria reportedly have negative attitudes towards using contraceptives to limit their total number of children but are willing to use them to increase birth spacing [12]. Clumsy and culturally-insensitive attempts to promote use of modern contraception in conservative Muslim cultures, such as in Bauchi, have led to negative connotations of “family planning” as an externally-driven, unwelcome concept [13, 14]. Failure to understand the local context can generate unexpected barriers for reproductive health programs [15].

Our recent trial tested universal home visits to pregnant women and their spouses in Toro LGA, in Nigeria’s Bauchi State [16]. The home visitors shared evidence about local risks for maternal and child health, that households could act on themselves. The home visits led to a significant improvement in maternal outcomes [17]. In collaboration with the Bauchi State government, we are developing a culturally safe module about prevention of *kunika* to include in the home visits. To inform the contents of this module, we carried out participatory research in Bauchi, using fuzzy cognitive mapping to systematize local knowledge about the causes and prevention of *kunika*. This paper describes the methods and findings of creating and analysing the cognitive maps. We include the step of producing summary maps to be validated by community groups. The summary maps are the substrate for discussion with local dialogue groups to guide the development of the *kunika* module in the home visits; we will report on this separately.

## Methods

### Setting

Bauchi State in north-eastern Nigeria is home to about five million people, with majority Hausa and Fulani ethnic groups. Most are Muslim, with a small Christian minority. Our study took place in urban and rural communities in six wards of Toro LGA, the largest LGA in the State.

The 2018 Nigerian Demographic and Health Survey [18] provides information about Bauchi State. Literacy is low: 26% of adult women and 48% of adult men have basic literacy. The median age at first sexual intercourse among women is 15.4 years, and 41% of teenagers have already begun childbearing. Polygamy is common: 22% of men have more than one wife and 47% of women have a co-wife. Women have an average of 7.2 children and men’s ideal number of children is 8.6. Short birth interval is common in Bauchi: in the five years to 2018, 66% of births happened within 36 months of a previous birth (median of 30.9 months). Just 6% of women use any method of contraception. Infant mortality is 69 per 1000, and among those children who survive the first year, 84 per 1000 die before turning five years old.

### Fuzzy cognitive mapping

We used fuzzy cognitive mapping to model local knowledge of factors causing *kunika*. This technique is a flexible, yet robust, tool to depict knowledge of causal relationships related to an outcome of interest [19]. The maps are directed graphs that use arrows to show participant assumptions of causal connections between factors (nodes) and the outcome. Participants indicate the strength of the causal links with numerical weights assigned to each arrow [20]. The maps describe structures of shared beliefs of participants using everyday language. Subsequent application of fuzzy logic uses mathematical language [21] to describe these structures as soft models of systems of concepts [22].

Fuzzy cognitive mapping was described in the 1980s [19], building on graph theory [23] and more recent developments of fuzzy logic [24]. The relative simplicity of creating the maps means they are not limited to sophisticated knowledge sources. Researchers have used fuzzy cognitive mapping to facilitate stakeholder engagement in environmental planning [25–27]. Giles pioneered the use of fuzzy cognitive mapping as an intercultural tool to facilitate communication between published evidence and indigenous knowledge [28], and Dion formalized this approach into the *Weight of Evidence* to contextualise the best available evidence according to the knowledge of relevant stakeholders [29].

Findings from our focus group discussions about *kunika* indicated that there were many reasons why people might continue to practise *kunika* even though they knew it had many adverse consequences. We chose to use fuzzy cognitive mapping to explore in more detail these causes of *kunika* for several reasons. It allows complex systems to be constructed by identifying one relationship (cause-arrow-outcome) at a time; this facilitates the discussion of complex systems even across language and cultural differences. Analysis of fuzzy cognitive maps indicates the relative strength of direct and indirect causes of the outcome, highlighting key factors to address for prevention. The maps provide a visual presentation of findings that can help to communicate the results, even to people with limited numeracy and literacy.

### Sampling and recruitment

We facilitated 26 mapping sessions. We stratified the communities in each of the six wards into urban, rural and rural remote groups and randomly selected two communities from each group (12 communities in total). Within these communities, men and women eligible to participate in the mapping groups were general community members, had been visited by the home visits program, were of reproductive age or older, married and with children. In each community, we facilitated a group of women and a separate group of men (24 groups in total). In some communities we held groups of younger men and women (aged less than 25 years) and in others we held groups of older men and women (aged 25 years and above). Each mapping group had eight or nine members. Team members from Bauchi State who had facilitated previous activities in these communities and were familiar with local customs and traditions visited each community to meet the village chief and council and discuss the activity with them. The community leaders contacted potential participants face to face and identified those who were eligible, available and willing to participate.

We also recruited a group of service providers (male and female together) who had worked with the team in the home visits project to participate in a mapping session at the LGA level. A group of health planners, working with the team on the home visits project, participated in a mapping session at the State level.

### Creating the maps

Two trained local researchers, fluent in Hausa, supported each mapping session. The community sessions took place in a neutral venue, often the school, and the LGA and State sessions took place in government offices. The facilitator guided the process and the reporter took notes of the discussion and the meanings of elements included in the maps. Male researchers facilitated male sessions and female researchers facilitated female sessions. Each group produced two maps: one of causes of *kunika* and one of causes of no*-kunika* (*Ba kunika* in Hausa) or preventive factors for *kunika*. We produced separate maps for causes and prevention of *kunika* to help focus the discussion in the groups.

Each mapping session followed the same procedure and lasted about three hours to produce two maps. The facilitator wrote the outcome (*kunika* or *ba kunika*) on a magnetic tile placed near the middle of a metal whiteboard. The facilitator invited participants in turn to mention causes of the outcome according to their knowledge, writing each cause on a tile and placing it on the board. If duplicate causes were mentioned, one mention was eliminated if all agreed it was really a duplicate. Many of the participants had limited or no literacy, and the facilitator read out what he or she wrote on each tile, and frequently reminded the participants about what was on each tile on the board. This was not a problem for the illiterate participants, as they are used to relying on their memory. When no additional concepts were mentioned, the groups identified relationships between factors and the facilitator drew arrows between concepts (nodes) to indicate these relationships. When the group had identified all the relationships on the map, the facilitator asked them to identify which one was the strongest arrow (weight 5) and which one was the weakest arrow (weight 1). Using these two weighted relationships as a reference, the group allocated weights to all the other relationships.

**Fig. 1:**
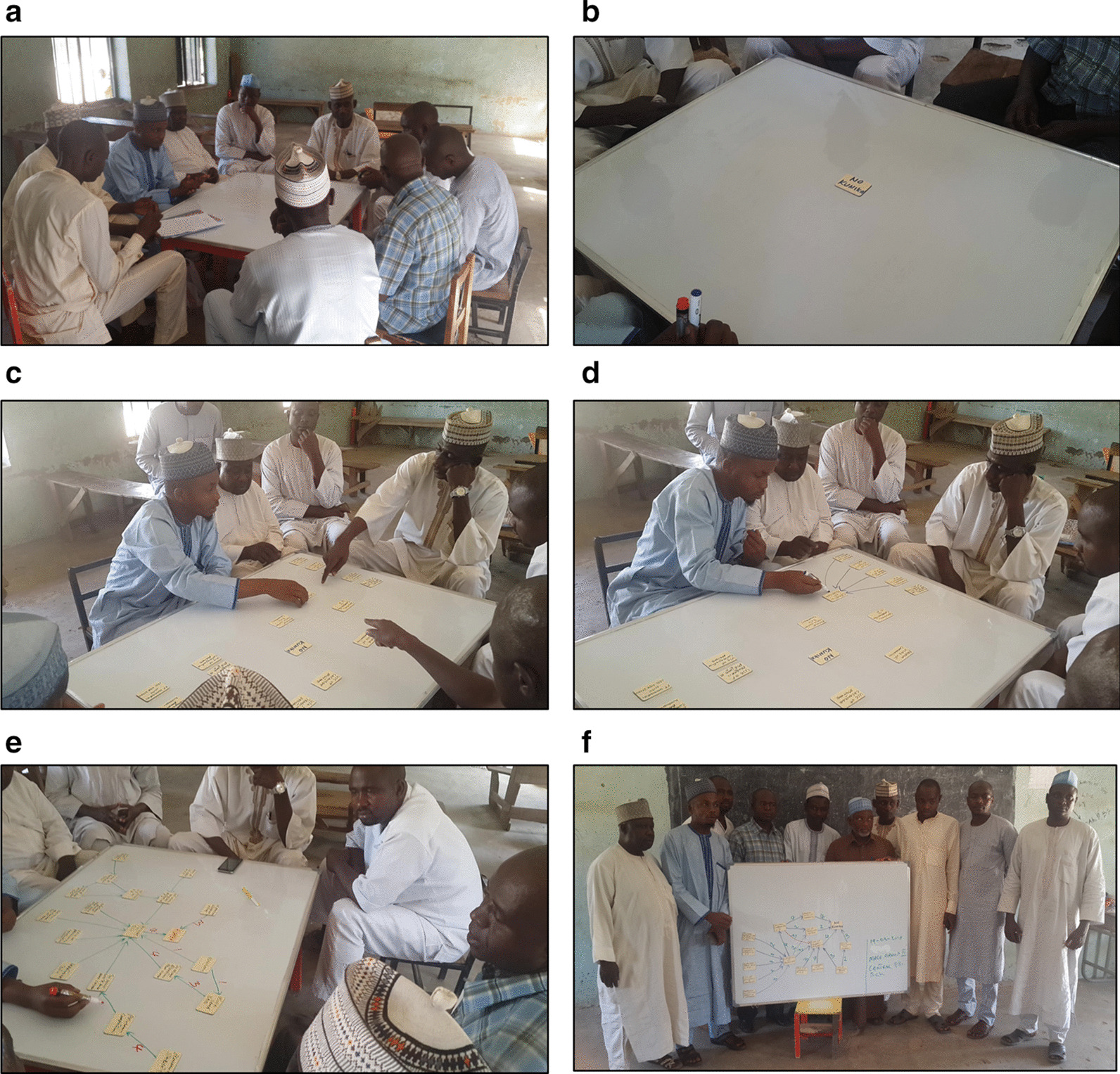
Steps to draw a fuzzy cognitive map in a group session. **a** Explain the process to the group. **b** Identify the outcome of interest. **c** Identify factors (nodes) causing the outcome. **d** Draw the relationships (arrows) linking the nodes. **e** Weight the strength of each relationship (1 to 5). **f** Verify the map with participants

At the end of the session, each group reviewed their map to confirm that it represented their views. The sequence of pictures in Fig. 1 summarizes the procedure. The groups made their decisions by consensus. Many groups had lively discussions, for example about the weights of different relationships, but all finally came to agreement without the need to vote.

### Analysis of the maps

We used freeware yEd [30] to digitize the maps. We then put the lists of nodes across all the maps into a table and standardized the naming, to avoid nodes with the same meaning having different wording. For example, we standardized nodes named “not using family planning”, “family planning not used”, “non-use of contraception”, “not using CP” as “not using family planning”. The notes from the mapping sessions helped to clarify the meaning of nodes when this was in doubt. Two authors (IS and AC) grouped individual factors into broader categories using an inductive approach[31] based on shared interpretation [32]. The fieldworkers who facilitated the mapping sessions and members of the local research team then reviewed and adjusted the standardized names of the nodes and the broader category groups.

With nodes in all maps in a standard format, we used fuzzy transitive closure in CIETmap, an open-source package providing an interface with the R statistical language, to convert each map into a knowledge network [33]. We then combined the maps in each stakeholder group, female and male groups separately, using the average of the weights in individual maps after transitive closure. Transitive closure identifies all the possible paths through which each factor might influence the outcome of interest and calculates the highest influence across those paths [34]. From the transitive closure analysis, we created condensed maps [26] of the most influential causal and protective categories for *kunika.* These maps reduced the number of factors into fewer categories, defined in the inductive thematic analysis. For the relationships between categories, we calculated the cumulative net influence as the sum of weights of the influences in the maps for that category over the maximum total cumulative weight in the condensed map. The cumulative net influence is a proportional score between 1 for the strongest relationships and 0 for those relationships without any influence. We created pattern matching tables [20] to compare the cumulative net influence of each category and the number of maps mentioning each category between male and female groups.

### Alternative to participant weighting

Participant weighting of each identified cause takes time, often doubling the duration of the map building session. In some settings, this is not possible due to time and other constraints. We tested an alternative operator-independent strategy by deleting the weights before combining the maps, allowing the mention of each causal link in an individual map to contribute to the weight in the compound map. Inspired by and analogous to discourse analysis following the original Harris approach [35], we treated each link between a causal factor and an outcome as a morpheme (an irreducible word, part of word or phrase that has meaning). By counting the frequency of each link across several maps, we could weight its perceived importance.

### Communicating about the maps

We created summary maps showing only the three strongest categories causing or preventing *kunika,* for all 48 community maps combined and for male and female maps separately*.* We labelled categories on these summary maps in Hausa and colour-coded the categories to help illiterate people review the maps. In a member checking exercise [36], local facilitators re-convened the groups of women and men who had built the original maps and presented the summary male, female, and overall community maps to them. They asked the participants if the summary maps fairly represented the main ideas in their original maps. The groups went on to suggest how to discuss the, often sensitive, issues in the maps with households in an acceptable way. We are preparing a separate report about these discussions and their use to co-design a module about *kunika* to include in our home visits to pregnant women and their spouses.

## Results

In total, 97 women and 100 men participated in 48 mapping sessions across six rural and six urban communities. All participants were married, with ages ranging from 18 to 60 years for women, and 22 to 78 years for men. One third of the women and one quarter of the men had no formal education; one quarter of the women and half the men had at least secondary education. We did not set out to document participant reactions to making fuzzy cognitive maps, but all facilitators noticed the active engagement of participants and their satisfaction with their maps. Creating the maps and seeing their knowledge reflected in this way was a validating experience for participants.

### Causes of *kunika*

**Fig. 2:**
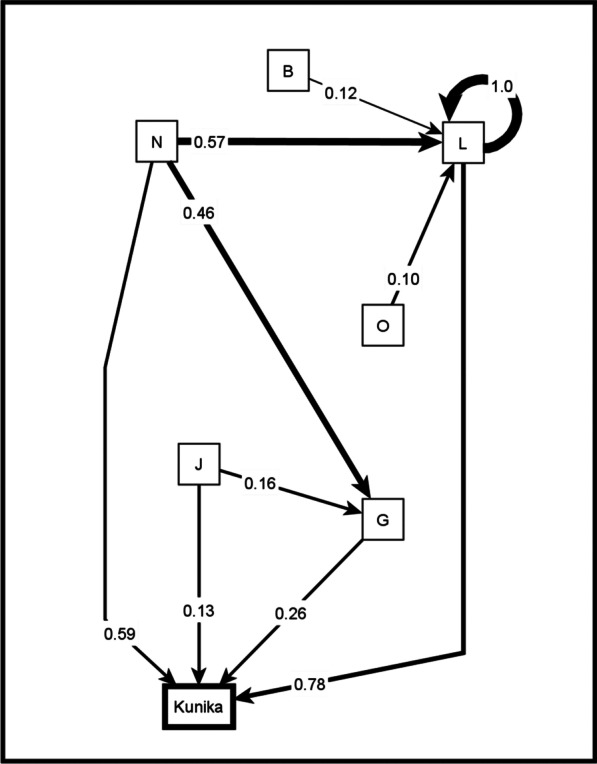
Composite fuzzy cognitive map of the causes of *kunika*. Composite map from 24 maps (12 female maps and 12 male maps). The weights of the arrows indicate the cumulative net influence. The thickness of the lines is proportional to the weights. Self-pointing arrows indicate that separate maps indicated internal dynamics of these categories, meaning that some factors within the category contribute to the occurrence of other factors in the same category. To simplify the map, we included only the relationships affecting the four strongest causes of *kunika*. B: Use of force or coercion; G: Not using modern methods of family planning; J: Lack of awareness about family planning and fear of side effects; L: Frequent sex and factors encouraging this; N: Family dynamics; O: Negative socio-economic conditions

**Fig. 3:**
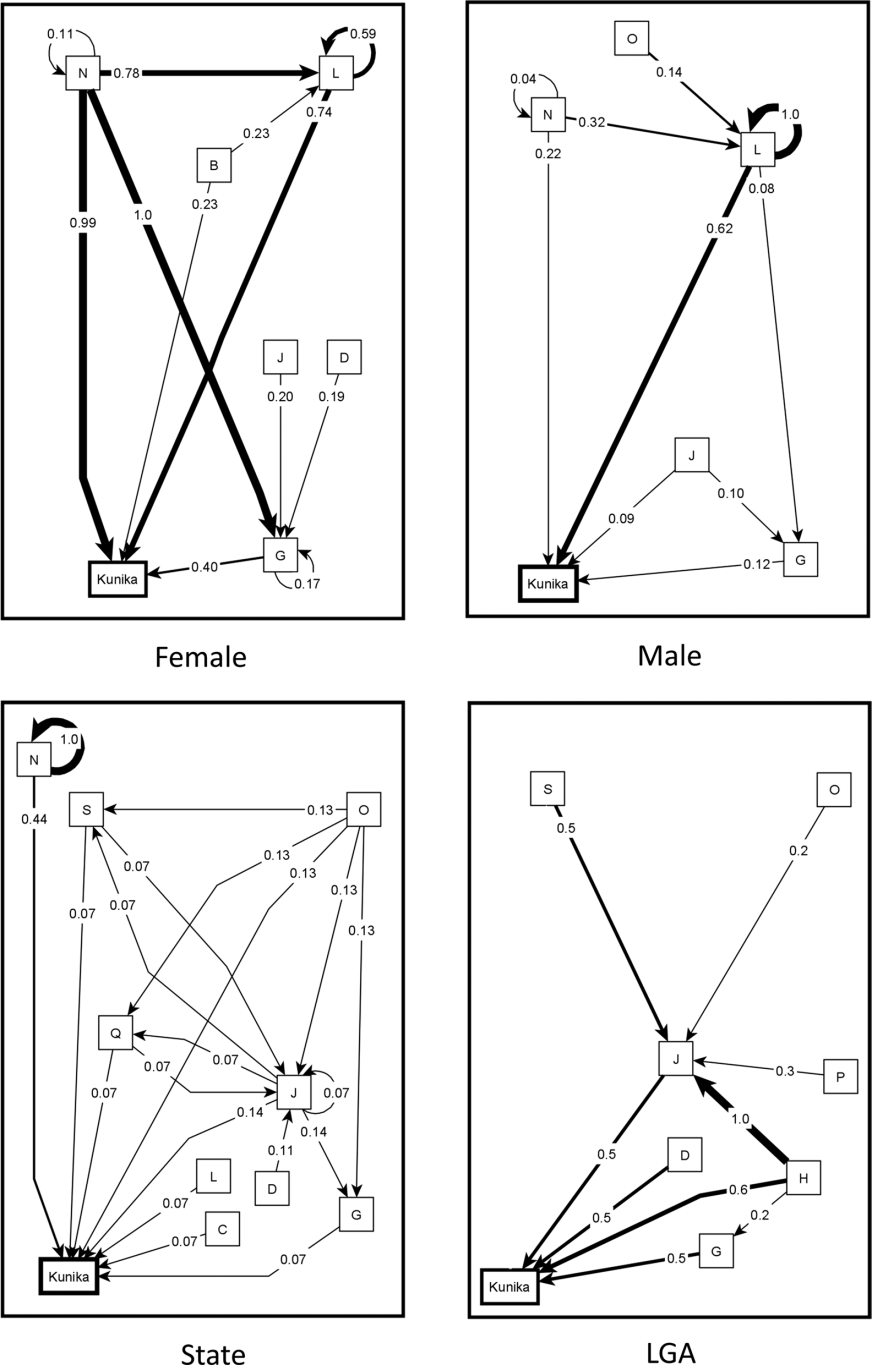
Separate maps of the causes of *kunika*. Female map synthesizes 12 female groups. Male map synthesizes 12 male groups. The State map and LGA map come from one group session each. The weights of the arrows indicate the cumulative net influence. The thickness of the lines is proportional to the weights. Self-pointing arrows indicate that separate maps indicated internal dynamics of these categories, meaning that some factors within the category contribute to the occurrence of other factors in the same category. To simplify the maps, we only included the relationships affecting the four strongest causes of *kunika*. B: Use of force or coercion; C: Lack of spousal communication about family planning; D: Lack of male involvement; G: Not using modern methods of family planning; H: Lack of use of health services; J: Lack of awareness about family planning and fear of side effects; L: Frequent sex and factors encouraging this; N: Family dynamics; O: Negative socio-economic conditions; P: Belief in faith and fate; Q: Lack of awareness about *kunika*; S: Lack of stigma around *kunika*

Across all groups, we identified a list of 101 unique causal factors for *kunika* and grouped these into 15 categories. Additional file 1 includes a table of all the unique factors across all the maps and the corresponding categories from the inductive analysis. Figure 2 shows the map of causes of *kunika* after transitive closure, for all community maps combined. Figure 3 shows the separate maps of males and females in communities, the map from LGA officers, and the map from State level officers. To facilitate their interpretation, the maps in Figs. 2 and 3 show only the relationships for the top four strongest causes. Additional file 2 includes a spreadsheet with all the relationships in the composite maps.

For all maps combined, the four most influential risk categories for *kunika* were:

1. *Frequent sex*: In addition to "frequent sex", this category included things leading to frequent sex, such as high desire, sharing a bed, the husband being present around the house, love and attraction, wearing make-up or tight clothes, sweet women, and handsome men. It also included the use of sex-drive medicines, pornography, carelessness and impatience. It included sexual contact while breastfeeding, and loss of the traditional practice of sending the wife to her parent’s house after the baby was born.
2. *Not using modern methods of family planning*: This category included not accepting modern family planning methods, refusal to have contraceptive injections, refusal to go to health facilities or health workers for contraception advice, and non-availability of family planning commodities.
3. *Family dynamics*: This complex category covered interactions between spouses and with other family members and social pressures and norms favouring short birth interval and/or large families. It included both monogamy, because all the reproductive load falls to one wife, and factors related to polygamy, including jealousy and competition between wives. It encompassed the desire for a large family and desire for a child of a particular sex. It covered women wanting to complete their family quickly, social and family pressure to have a large family, and women wanting to satisfy their husbands so that they remain faithful.
4. *Not using non-invasive family planning methods*: This included not using condoms, nor the withdrawal method, nor calendar method, nor traditional methods and remedies. Some groups stressed that men refused to use these methods.

The pattern-matching table (Table 1) highlights differences in perspectives of stakeholder groups about causes of *kunika*. Women and men coincided in the first three most influential categories in all maps, based on cumulative net influence, although the order of the three categories differed. For women’s groups, *family dynamics* was the most influential category, followed by *frequent sex*. The fourth most influential category identified by women was *use of force or coercion*: this included men forcing women to have sex, male dominance, and women coercing men to have sex.

**Table 1:** Pattern matching table of causes of *kunika*

Categories of causes of *kunika*	Composite map		Women’s maps		Men’s maps		LGA maps		State maps	
	CNI	# of maps	CNI	# of maps	CNI	# of maps	CNI	# of maps	CNI	# of maps
*Total maps*	-	24	-	12	-	12	-	1	-	1
Frequent sex and factors encouraging this	0.78 (1)	24	0.74 (2)	12	0.62 (1)	12	0.40	1	0.07	1
Family dynamics	0.59 (2)	24	0.99 (1)	12	0.22 (2)	12	0.15	1	0.44 (1)	1
Not using modern methods of family planning	0.26 (3)	23	0.40 (3)	12	0.12 (3)	11	0.50 (2)	1	0.07	1
Lack of awareness about family planning and fear of side effects	0.13 (4)	20	0.15	11	0.09 (4)	9	0.50 (2)	1	0.14 (2)	1
Use of force or coercion	0.11	14	0.23 (4)	11	0.02	3	0.25	1	0.04	1
Not using non-invasive family planning methods	0.11	16	0.18	10	0.04	6	0.00	0	0.00	0
Lack of use of health services	0.08	16	0.07	7	0.06	9	0.60 (1)	1	0.00	1
Negative socio-economic conditions	0.07	11	0.03	2	0.08	9	0.10	1	0.13 (3)	1
Belief in faith and fate	0.07	15	0.08	7	0.05	8	0.15	1	0.06	1
Ineffective family planning	0.06	7	0.10	5	0.03	2	0.00	0	0.00	0
Lack of awareness about *kunika*	0.06	8	0.03	1	0.06	7	0.25	1	0.07	1
Lack of male involvement	0.05	11	0.12	11	0.00	0	0.50 (2)	1	0.06	1
Fertility, including fertility after giving birth	0.05	10	0.09	7	0.01	3	0.15	1	0.07	1
Lack of spousal communication about family planning	0.04	7	0.02	2	0.03	5	0.20	1	0.07	1
Lack of stigma around *kunika*	0.01	3	0.00	0	0.02	3	0.35	1	0.07	1

The numbers in brackets indicate the ranking for the first four most influential categories for each stakeholder group

CNI: cumulative net influence on *kunika*

Men’s maps identified *frequent sex* as the category with by far the highest influence, followed by *family dynamics*. The composite male map had in the fourth position *lack of awareness about family planning and fear of side effects*: this included statements about fear of complications of birth control methods, and fear of specific complications (like contraceptives making women infertile).

*Lack of male involvement* appeared in 11 women’s maps but in none of the men’s maps. Similarly, *use of force or coercion* was mentioned in 11 women’s maps but in only three men’s maps. On the other hand, *lack of awareness about kunika* rarely appeared in women’s maps (1 map) but was more frequent in men’s maps (7 maps). The concern for the role of *negative socio-economic conditions* as a cause of *kunika* was also less frequent among women (2 maps) than it was among men (9 maps).There were differences between health providers at the LGA level and men and women in communities; the service providers emphasized lower use of health services, lack of use and awareness of family planning methods, and lack of male involvement. Only the State map included negative socioeconomic conditions among the four most important categories of causes of *kunika*.

### Causes of no-*kunika* (protective factors)

**Fig. 4:**
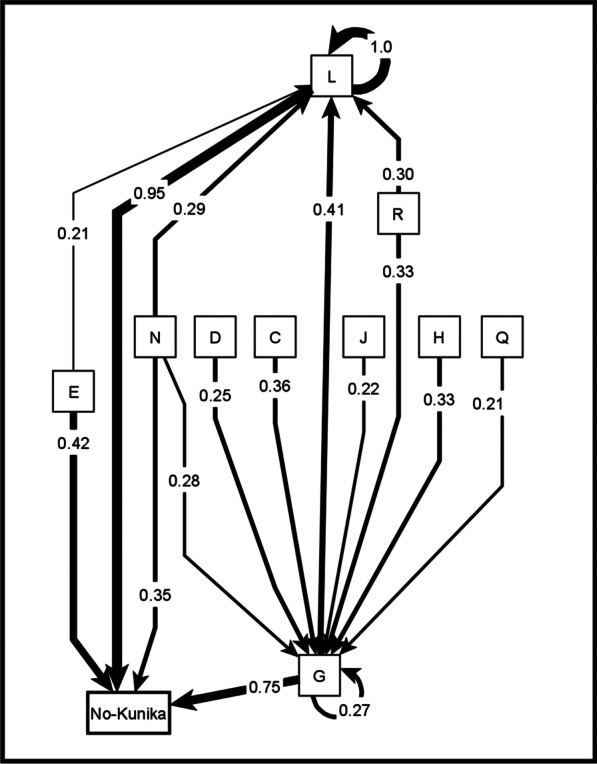
Composite fuzzy cognitive map of the protectors (causes of no-*kunika*). Composite map from 24 maps (12 female maps and 12 male maps). The weights of the arrows indicate the cumulative net influence. The thickness of the lines is proportional to the weights. Self-pointing arrows indicate that separate maps indicated internal dynamics of these categories, meaning that some factors within the category contribute to the occurrence of other factors in the same category. To simplify the map, we included only the relationships affecting the four strongest causes of no-*kunika*. C: Better spousal communication about family planning; D: Greater male involvement; E: Using non-invasive family planning methods; G: Using modern methods of family planning; H: Use of health services and advice from health workers; J: Increase awareness about family planning; L: Reduce frequent sex; N: Family dynamics; Q: Increase awareness about *kunika*; R: Create public awareness about family planning and *kunika*

**Fig. 5:**
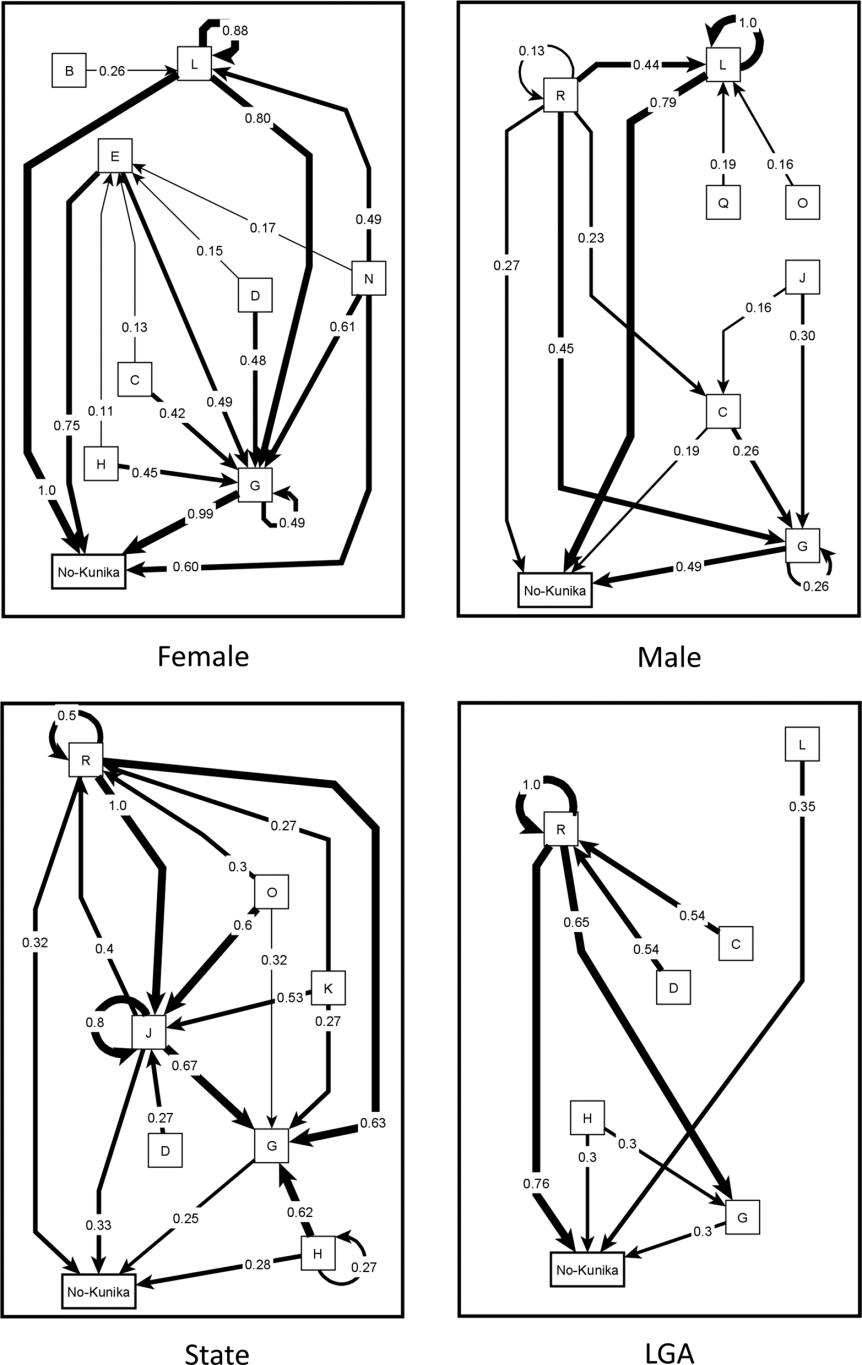
Separate maps of protectors (causes of no-*kunika*). Female map synthesizes 12 female groups. Male map synthesizes 12 male groups. The State map and LGA map come from one group session each. The weights of the arrows indicate the cumulative net influence. The thickness of the lines is proportional to the weights. Self-pointing arrows indicate that separate maps indicated internal dynamics of these categories, meaning that some factors within the category contribute to the occurrence of other factors in the same category. To simplify the maps, we included only the relationships affecting the four strongest causes of no-*kunika*. B: Prevention of forced sex; C: Better spousal communication about family planning; D: Greater male involvement; E: Using non-invasive family planning methods; G: Using modern methods of family planning; H: Use of health services and advice of health workers; J: Increased awareness about family planning; K: Ways of increasing family planning awareness; L: Reduce frequent sex and factors encouraging this; N: Family dynamics; O: Better socio-economic conditions; Q: Increase awareness about *kunika*; R: Create public awareness about family planning and *kunika*

In the composite map of protective factors, we found 98 unique protective factors and grouped these into 18 categories. Additional file 2 presents the table with all the unique factors across all the maps and the corresponding categories from the inductive analysis. Figure 4 shows the map of protective factors for *kunika* arising from the transitive closure, for all the community maps combined. Figure 5 shows the separate maps for the males, females, LGA officers, and State level officers. To facilitate their interpretation, the maps in Figs. 4 and 5 show only the relationships for the top four strongest causes. Additional file 3 shows the list of all relationships identified in the maps of protective factors.

The four main protective factors in the overall map (Table 2) reflected the inverse of the main causal factors for *kunika* (Table 1).

1. *Reduce frequent sex*: This category included statements like staying away from the husband while breastfeeding, and advice to avoid sex for 4–5 months after delivery.
2. *Using modern methods of family planning*: This included using various modern methods of family planning and taking advice about this from health workers. It also included increasing demand for family planning (for example, by advice from health workers), increasing availability of family planning commodities, free distribution of commodities, and developing commodities with less side-effects.
3. *Using non-invasive family planning methods*: This included using condoms, withdrawal method, calendar method, and traditional methods.
4. *Family dynamics*: Polygamy was a protective factor, since the burden of bearing children is shared between co-wives. This category also included finding ways to avoid competition between co-wives and reducing the demand for large families.

**Table 2:** Protective factors for *kunika*

Categories of causes of no-*kunika*	Composite map		Women’s maps		Men’s maps		LGA maps		State maps	
	CNI	# of maps	CNI	# of maps	CNI	# of maps	CNI	# of maps	CNI	# of maps
Total maps	–	24	–	12	–	12	–	1	–	1
Reduce frequent sex	0.95 (1)	24	1.00 (1)	12	0.79 (1)	12	0.35 (2)	1	0.08	1
Using modern methods of family planning	0.75 (2)	24	0.99 (2)	12	0.49 (2)	12	0.30 (3)	1	0.25 (4)	1
Using non-invasive family planning methods	0.42 (3)	20	0.75 (3)	12	0.13	8	0.05	1	0.00	0
Family dynamics	0.35 (4)	22	0.60 (4)	12	0.13	10	0.00	0	0.05	1
Better spousal communication about family planning	0.24	8	0.26	7	0.19 (4)	1	0.14	1	0.15	0
Use of health services and advice from health workers	0.22	8	0.28	2	0.14	6	0.30 (3)	1	0.28 (3)	1
Create public awareness about family planning and *kunika*	0.20	20	0.05	11	0.27 (3)	9	0.76 (1)	1	0.32 (2)	1
Greater male involvement	0.17	11	0.26	2	0.09	9	0.14	0	0.07	1
Increase awareness about *kunika*	0.16	14	0.16	7	0.13	7	0.14	1	0.08	1
Increase awareness about family planning	0.15	16	0.07	9	0.19	7	0.00	1	0.33 (1)	1
Effective family planning	0.14	22	0.18	11	0.09	11	0.00	1	0.00	1
Better socio-economic conditions	0.11	10	0.13	7	0.08	3	0.11	0	0.17	0
Prevention of forced sex	0.10	1	0.21	1	0.01	0	0.03	0	0.00	0
Reducing fertility after birth	0.04	2	0.09	2	0.00	0	0.11	0	0.02	1
Ways of increasing family planning awareness	0.03	5	0.07	5	0.00	0	0.00	1	0.13	1
Increase stigma about *kunika*	0.02	7	0.00	3	0.03	4	0.00	1	0.00	1
Seek advice from traditional birth attendants	0.01	1	0.03	1	0.00	0	0.00	0	0.00	0
Belief in faith and fate	0.00	3	0.01	0	0.00	3	0.00	0	0.00	0

CNI: cumulative net influence on no-*kunika*

The numbers in brackets indicate the ranking for the first four most influential categories for each stakeholder group

As shown in Table 2, there were differences between men and women. Men and women agreed about the importance of *reducing frequent sex* and *using modern methods of family planning*. Women’s maps also highlighted *using non-invasive methods of family planning* and *changing family dynamics*, while men’s maps, as well as the maps of service providers and state-level decisions makers, emphasized *creation of public awareness about family planning*.

### Alternative to participant weighting

Operator-independent weighting based on the Harris approach to discourse analysis proved to be an effective substitute for participant weighting. Using the existence of the link (yes/no) across each of 12 maps per stakeholder group and counting the frequency of occurrence produced essentially the same results as participant weighting (see Additional file 4).

### Member checking of summary maps

**Fig. 6:**
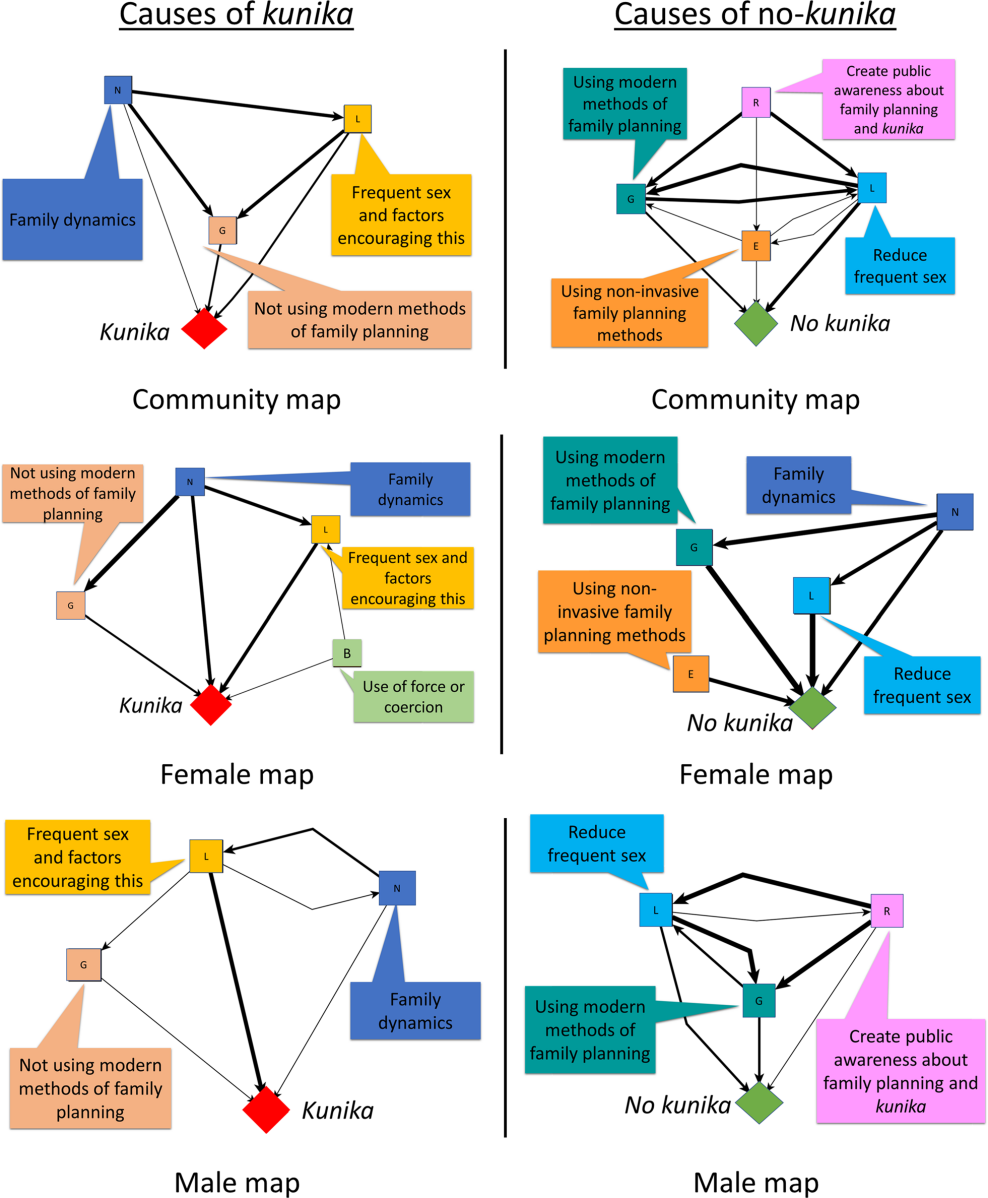
Summarized maps for sharing with community dialogue groups. Female map synthesizes 12 female groups, male map synthesizes 12 male groups, and community map synthesises all of them. The thickness of the lines is proportional to the cumulative net influence. To simplify the graph, we only included the relationships affecting the three strongest causes of *kunika*. The original labels of the maps were in Hausa language. Colour coding helps interpretation by illiterate participants

Figure 6 shows the summary maps we created for discussion in communities. The community groups of women and men who built the original maps found the summary maps relatable. Participants were able to explain what the maps represented. None of the groups disagreed with the categories combining groups of causes and all groups considered that the maps were a fair representation of their knowledge about causes of *kunika*.

## Discussion

Men and women in communities in Bauchi readily identified a range of factors they considered causal and preventive for *kunika* and indicated their views about the strength of the links between these factors and the outcome. They identified the most influential causes of *kunika* as frequent sexual contact, family dynamics, and non-use of both modern and traditional contraceptive methods. In separate maps of factors protecting against *kunika* they mainly identified as most influential the mirror-images of these causes.

Women and men had some different beliefs. Women, but not men, recognised forced or coerced sex as an important cause of *kunika*. Nearly all the women’s maps, but none of the men’s maps, mentioned lack of male involvement in reproductive health as a cause of *kunika*. On the other hand, men’s maps, and those of health providers at LGA and State level, placed more emphasis on increasing awareness about *kunika* and family planning methods as a way of reducing *kunika*.

The causes of *kunika* identified by women and men in Bauchi communities differ from those reported to be associated with short birth interval in quantitative studies in low- and middle-income countries. In a recent systematic review [11] only two factors were consistently associated with short birth interval in quantitative studies: shorter duration of breastfeeding and female sex of the previous child. The factors most frequently examined in the reviewed studies included age of the mother, education of the mother, education of the father, and household socio-economic status. Some of the maps in our study mentioned socio-economic status as a factor, but not a strongly influential factor. Age of the mother and education of the mother did not feature at all in the maps, perhaps because in the Bauchi context young age of mothers and low education of women are the norm, and do not explain why some women have *kunika* and others do not.

All groups in Bauchi identified frequent sex, with factors leading to this, as a key cause of *kunika*. Some maps specifically identified early resumption of sexual intercourse during breastfeeding as a cause of *kunika*. Qualitative studies, in Nigeria and elsewhere, have explored the dynamics of sexual contact after the birth of a child and during the period of breastfeeding; some have pointed to abandonment of traditional practices and taboos during this period as a cause of short birth interval [37–39]. Some of the dynamics around sexual intercourse between couples might have changed with the cultural transition towards more modern lifestyles and consequent changes in conservative sexual behaviour and social taboo [37, 38]. A study in the south of Nigeria found that loss of traditional healthcare practices was associated with more western education [40]. However, most studies examining maternal education and birth interval have found shorter birth interval associated with less maternal education [11]. The mapping groups in Bauchi talked openly about the use of aphrodisiacs and pornography as causes of frequent sex and hence *kunika*. Use of herbal aphrodisiacs is reportedly common among women in northern Nigeria [41, 42]. We are not aware of any other study reporting that men and women link the use of aphrodisiacs to *kunika*.

Participants identified use of contraception as influential in reducing *kunika*, counteracting the influence of frequent sex. Most studies reporting an association between contraceptive use and birth interval in low- and middle-income countries have found a longer birth interval among women using contraception [11]. One multi-country analysis of data from Demographic and Health Surveys found shorter birth interval associated with modern contraceptive use, possibly because women began using contraception after experiencing an unintended short birth interval [43]. Notes from the sessions discussing the summary maps in Bauchi indicated that participants felt people would not be willing to reduce sexual contact but would be interested to use contraception to make such contacts less likely to lead to *kunika*. Focus group discussions with women in Nigeria suggested that they rated the risks of short birth interval as greater than the risks of modern temporary methods of contraception [44].

All the mapping groups identified family dynamics as an important cause of *kunika* and identified modification of these dynamics as crucial to tackling *kunika*. These dynamics help to explain why people may continue to practise *kunika*, even when they know it has adverse consequences [10]. The category includes the interaction between spouses (including interactions between co-wives), motivations to have more children and the influence of extended family and neighbours. Decisions about child spacing and family size are complex, and not well-reflected by the narrow concept of “unmet need for contraception”. Simply urging women to use contraception, without considering conflicting family dynamics, is unlikely to succeed.

Other studies in Nigeria have reported family dynamics that favour a short birth interval, such as a desire to have a large family, the need for children for a workforce, the influence of in-laws, and uncertainty surrounding children’s survival [45, 46]. Children as a source of male pride and competition between co-wives may favour short birth interval in Kenya [38]. We considered “desire for a child of a particular sex” as part of the category of family dynamics. This was often, although not always, a desire to have a male child when the preceding child was a girl. Previous studies in Ethiopia reported a shorter birth interval if the previous child was female [47, 48], and this was also reported in some ethnic groups in Nigeria [46].

Forced or coerced sex featured as an influential cause of *kunika* in maps created by women, but not those created by men. Notes from the mapping groups suggested that often the women began by mentioning “love” as a cause of *kunika*, but later discussed that women could not refuse sex with their husbands and faced violence if they tried to. Levels of violence against women remain high in Nigeria and are higher than the national average in Bauchi [18]. Less than half of women in Bauchi can refuse to have sex with their spouses [18]. A recent multicounty study based on data from Demographic and Health Surveys found that emotional, physical, or sexual intimate partner violence was associated with shorter interpregnancy intervals and more unintended pregnancies [49].

Although not identified as having the strongest influence on *kunika*, “lack of male involvement” featured as a cause of *kunika* in 11/12 of the women’s maps of causes of *kunika*; but it was not present in any of the men’s maps (Table 1). This category included men not being willing to use contraception or get advice about it, and not letting their wives get such advice. In many African countries men are traditionally the main decision makers about when to have sex, whether to use contraception [50, 51], and how many children to have [52], although in recent years joint decision about contraception and fertility preferences has become more common [53]. On the other hand, men’s maps gave more prominence to increasing awareness about *kunika* and family planning as a way of reducing *kunika*. This may suggest that they are aware of their lack of knowledge about these issues and are willing to get more involved.

Building and analysing fuzzy cognitive maps was part of our participatory research [54] into *kunika* in Bauchi State. Our partnership with community members, service providers, and health planners began by hearing the views and knowledge of community members through focus groups [10] and the fuzzy cognitive mapping described here. It went on to sharing the knowledge between stakeholders, and to proposing and implementing solutions. Our work on *kunika* was stimulated by the Bauchi State health authorities who expressed a concern about lack of birth spacing and suggested an exploration of how prevention of *kunika* could be incorporated into the home visits program. They are now planning to expand the home visits, including the *kunika* module, across the State.

Future research using fuzzy cognitive mapping could examine further the use of operator-independent methods for weighting of identified causes, which our findings suggest is a promising approach. It would also be interesting to examine in more detail the intermediate causes and common pathways by which different factors impact the outcome of *kunika*.

### Limitations

Fuzzy cognitive mapping does not mean the causes identified are “true” causes of the outcome, though they are true causes in the understanding of those who built the maps. The concern of soft models, however, is to engage stakeholders in identifying how to tackle the issue of concern, in this case, *kunika*. For the participants, these maps offer a way to present and to reflect on what they know. For researchers, the maps are sources of new hypotheses and variables to make sense of the issue.

All the participants in the mapping groups were married with children and most of them had at least two children but having two or more children was not an eligibility requirement. The participants shared their knowledge about the causes of *kunika* based not only on their own experience but also on what they knew of the experience of family and other community members.

We used transitive closure to calculate the influence of causal categories on the outcome. The compound maps present complex networks of interactions and it is difficult to consider all the reported relationships. The output of transitive closure can seem complex, even to researchers, so it was important to create summary maps that community groups can readily review and use. Our summary maps focussed on the strongest categories, and this simplification risks losing information.

Fuzzy cognitive mapping collects a lot of information in a short time. In our case, creating one map for causes and one for protective factors increased the time needed with each group. We had a break for lunch between the two maps, but the participants may have been tired and less engaged when creating the second map. Given the similar results using Harrisian discourse analysis, future mapping sessions might leave out the step of participant weighting which, though informative, can take as long as the building of the map.

It was not feasible to return to the communities and engage the map authors in the thematic analysis. The researchers who defined the broader categories of causes and protective factors used the mapping session notes and knowledge of the local fieldworkers to clarify the meaning of concepts. They checked their categories with the local research team and group facilitators. The assumptions and implications of our categorization are explicit in Additional file 1. The community groups presented with the summary maps agreed these maps fairly represented their views.

## Conclusions

Fuzzy cognitive mapping by community members in Bauchi State confirms that *kunika* results from a complex network of interacting factors. These include culture-specific dynamics that external stakeholders often ignore. Although the promotion of contraception has a role to play in childbirth spacing, strategies relying only on this are likely to be insufficient.

This study demonstrates how communities can have a role in reducing *kunika*. The emphasis is not on knowing the true causes of *kunika,* but on supporting communities to identify what they know and what they can do to address the issue. Outputs of analysis of fuzzy cognitive maps can be made accessible to ordinary stakeholders, allowing their meaningful participation in interpretation and use of the findings.

## Supplementary Information

**Additional file 1.** Matching table of factors and categories across all the maps. A table presenting all the factors with similar meanings in the same row and the corresponding category assigned during the thematic analysis. The first two columns indicate the names of categories and standardised names of the factors. The remaining columns contain the factors in the corresponding map. There are two sheets, one for causes and another for protectors.

**Additional file 2.** Adjacency matrices of composite maps of risks by group. Each sheet contains the adjacency matrix of the corresponding map. Each cell in the adjacency matrix indicates the weight of a relationship between two categories. The rows are the initial category and the columns the landing one for each relationship.

**Additional file 3.** Adjacency matrices of composite maps of protectors by group. Each sheet contains the adjacency matrix of the corresponding map. Each cell in the adjacency matrix indicates the weight of a relationship between two categories. The rows are the initial category and the columns the landing one for each relationship.

**Additional file 4.** Pattern matching table to compare participant weighted and discourse analysis weighted maps. There is one table for each composite map of risks and protectors (communities, female and male groups). Each table contains two columns indicating the cumulative net influence of the category on *kunika* or no-*kunika* from the original maps or from the discourse analysis. For each category, we indicated the order relative to all the categories influencing the outcome in the map.

**Additional file 5.** Full text in Spanish version.

## Data Availability

The data that support the findings of this study are available from the authors upon reasonable request. Data were used for the current study under authorization from participating communities. Therefore, sharing the data will require permission from the participating communities.

## Abbreviations

CNI: Cumulative Net Influence
LGA: Local Government Area
WHO: World Health Organization
