# Supplementary material for: Causes of short birth interval (kunika) in Bauchi State, Nigeria: systematizing local knowledge with fuzzy cognitive mapping
Source: Reprod Health. 2021 Apr 6;18:74. doi: 10.1186/s12978-021-01066-2 (PMC8022364; doi:10.1186/s12978-021-01066-2)
Supplement: Supplementary file 5 — Additional file 5. Full text in Spanish version. [file 12978_2021_1066_MOESM5_ESM.pdf]

Reproductive Health

RESEARCH Open Access

Causas del intervalo intergenésico corto (*kunika*) en el estado de Bauchi, Nigeria: sistematización del conocimiento local con mapeo cognitivo

Ivan Sarmiento[1] *, Umaira Ansari[2], Khalid Omer[2], Yagana Gidado[3], Muhammad Chadi Baba[3], Adamu Ibrahim Gamawa[4], Neil Andersson[1,2], and Anne Cockcroft[1,2]

RESUMEN

Antecedentes: Los intervalos intergenésicos cortos (menores de 33 meses, según la OMS) afectan la salud y el bienestar de la madre, el niño y la familia. Aunque los habitantes del norte de Nigeria reconocen muchos efectos adversos de un intervalo intergenésico corto (*kunika* en lengua hausa), éstos aún son frecuentes. Nosotros usamos cartografía cognitiva para sintetizar el conocimiento local sobre causas de *kunika* y guiar el codiseño de estrategias culturalmente seguras que permitan su disminución.

Método: Grupos de hombres y mujeres en doce comunidades hicieron 48 mapas cognitivos, y funcionarios del Estado de Bauchi y del Área Local de Gobierno (LGA por sus siglas en inglés) hicieron otros cuatro. Cada mapa mostraba causas de *kunika* o de *no-kunika* con flechas indicando la influencia entre ellas. Los participantes ponderaron la influencia entre 1 (la más débil) y 5 (la más fuerte). Nosotros combinamos los mapas por grupos de mujeres, hombres y funcionarios. Con *fuzzy transitive closure* calculamos la máxima influencia entre factores cuando todas relaciones en el mapa son consideradas. Condensamos los mapas agrupando factores individuales en categorías y calculamos la influencia neta acumulativa para cada una. Estos mapas los sintetizamos aún más para revisarlos con sus autores.

Resultados: Los mapas de los grupos comunitarios identificaron el sexo frecuente y factores relacionados, no usar anticonceptivos modernos o tradicionales y las dinámicas familiares (como el deseo de tener más hijos o la competencia entre esposas) como las causas más importantes de *kunika*. Los mapas de las mujeres identificaron el sexo forzado como causa importante y los mapas de los hombres destacaron la falta de conocimiento sobre anticoncepción y el temor a los efectos secundarios. La falta de participación masculina apareció en los mapas de las mujeres, los funcionarios del LGA y del Estado. Los mapas de los factores protectores correspondieron en gran medida a los opuestos de las causas. Los grupos comunitarios aprobaron y apreciaron los mapas que sintetizaban el análisis.

Conclusiones: Los mapas comunitarios mostraron que *kunika* es el resultado de una compleja red de factores con dinámicas culturales específicas. Es poco probable que enfocarse solo en promover anticoncepción reduzca *kunika*. Los resultados del *transitive closure* pueden comunicarse al público en general para una mayor participación en la interpretación y uso de los resultados.

Palabras clave: Intervalo intergenésico, mapeo cognitivo, diálogo deliberativo, salud reproductiva, [illegible]

* Autor de correspondencia: Iván Sarmiento
ivan.sarmiento@mail.mcgill.ca
Department of Family Medicine - McGill University
La lista con la información de los autores esta al final del artículo

Antecedentes

Un grueso cuerpo de literatura vincula el intervalo intergenésico corto con la morbilidad y la mortalidad infantil [1, 2]. Un análisis sugirió que evitar los intervalos intergenésicos de menos de 36 meses podría haber evitado el 35% de las muertes entre los niños menores de cinco años en los países en desarrollo [3]. La Organización Mundial de la Salud recomienda un intervalo entre un parto y el siguiente embarazo de al menos 24 meses, equivalente a un intervalo intergenésico de aproximadamente 33 meses [4]. Un análisis reciente de los datos de la Encuesta Demográfica y de Salud (DHS) de 77 países reportó que los riesgos para la salud infantil de un intervalo intergenésico corto se encuentran principalmente en los países de ingresos bajos y medios [5]. La evidencia de las consecuencias adversas para la salud materna del intervalo intergenésico corto es menos consistente [6]. Los mecanismos causales del impacto del intervalo intergenésico corto en la salud materna e infantil no se comprenden completamente [7]. Algunas asociaciones entre intervalos intergenésicos cortos y resultados adversos podrían deberse a factores de confusión (*confounders*) por factores socioeconómicos [8].

El intervalo intergenésico corto es un fenómeno bien reconocido en el norte de Nigeria; la palabra *kunika* en el idioma hausa significa quedar embarazada antes de que se destete al último niño. El norte de Nigeria es predominantemente musulmán y la enseñanza islámica recomienda amamantar durante 24 meses, [9] por lo que evitar *kunika* se alinea estrechamente con la recomendación de la OMS de un intervalo de nacimiento a embarazo de no menos de 24 meses. Los eruditos islámicos en Nigeria desalientan la *kunika* debido a sus riesgos para la salud de la madre y el niño [9]. Nuestras recientes discusiones de grupos focales comunitarios en el Área de Gobierno Local de Toro (LGA) en el estado de Bauchi, en el norte de Nigeria, confirmaron que los hombres y las mujeres tenían una buena comprensión del concepto de *kunika* y lo consideraban algo malo, que amenazaba la salud y el bienestar de madres, hijos y padres. Los participantes del grupo también describieron cómo la *kunika* podría conducir a dificultades económicas, conflictos dentro de las familias y desaprobación social [10].

Una revisión sistemática reciente examinó la evidencia cuantitativa y cualitativa sobre los factores relacionados con el intervalo intergenésico corto. Para la mayoría de los factores, la evidencia de los estudios cuantitativos fue mixta; sólo una menor duración de la lactancia materna y tener una niña en el parto anterior se asociaron sistemáticamente con un intervalo intergenésico corto [11]. Los estudios cualitativos incluidos en la revisión exploraron una gama más amplia de factores, incluida la comunicación entre parejas y familias, los medios de comunicación locales, la influencia social y el ejemplo de otros padres [11].

Según algunos reportes, los hombres del norte de Nigeria tienen actitudes negativas hacia el uso de anticonceptivos para limitar el número total de hijos, pero están dispuestos a usarlos para aumentar el espaciamiento de los nacimientos [12]. Los intentos torpes y culturalmente insensibles de promover el uso de anticonceptivos modernos en culturas musulmanas conservadoras, como en Bauchi, han generado a connotaciones negativas de la "planificación familiar" como un concepto indeseado e impulsado desde el exterior [13, 14]. No comprender el contexto local puede generar barreras inesperadas para los programas de salud reproductiva [15].

Nuestro ensayo controlado aleatorizado recientemente evaluó las visitas domiciliarias universales a mujeres embarazadas y sus cónyuges en Toro LGA, en el estado de Bauchi en Nigeria [16]. Los visitadores domiciliarios compartieron evidencia sobre los riesgos locales para la salud materna e infantil que los

hogares podrían usar para definir acciones autónomas. Las visitas domiciliarias resultaron en un mejoramiento significativo de los resultados de salud materna [17]. En colaboración con el gobierno del estado de Bauchi, estamos desarrollando un módulo culturalmente seguro sobre la prevención de *kunika* para incluirlo en las visitas domiciliarias. Para informar los contenidos de este módulo, realizamos una investigación participativa en Bauchi, utilizando el mapeo cognitivo para sistematizar el conocimiento local sobre las causas y la prevención de la *kunika*. Este artículo describe los métodos y hallazgos para crear y analizar los mapas cognitivos. Incluimos el paso de producir mapas de resumen, todavía para ser validados por grupos comunitarios. Los mapas de resumen son el sustrato de discusión con los grupos de diálogo locales para orientar el desarrollo del módulo *kunika* en las visitas domiciliarias; informaremos sobre esto por separado.

Métodos

Población del estudio

El estado de Bauchi en el noreste de Nigeria tiene a unos cinco millones de residentes, en su mayoría de los grupos étnicos Hausa y Fulani. La mayoría son musulmanes, con una pequeña minoría cristiana. Nuestro estudio se llevó a cabo en comunidades urbanas y rurales en seis distritos del Área de Gobierno Local (LGA) de Toro, el LGA más grande del estado.

La Encuesta Demográfica y de Salud de Nigeria de 2018 [18] proporciona información sobre el estado de Bauchi. La alfabetización es baja. El 26% de las mujeres adultas y el 48% de los hombres adultos tienen alfabetización básica. La edad media de la primera relación sexual entre las mujeres es de 15.4 años y el 41% han comenzado a tener hijos antes de los 20 años. La poligamia es común. El 22% de los hombres tiene más de una esposa y el 47% de las mujeres tiene una co-esposa. Las mujeres tienen un promedio de 7.2 hijos y el número ideal de hijos de los hombres es 8.6. El intervalo *intergenésico* corto es común en Bauchi; en los cinco años hasta 2018, el 66% de los nacimientos ocurrieron dentro de los 36 meses posteriores a un nacimiento anterior (mediana de 30.9 meses). Solo el 6% de las mujeres usa algún método anticonceptivo. La mortalidad infantil es de 69 por 1000, y entre los niños que sobreviven al primer año, 84 por 1000 mueren antes de cumplir los cinco años.

Mapeo cognitivo

Usamos el mapeo cognitivo para modelar el conocimiento local de los factores que causan *kunika*. Esta técnica es una herramienta flexible, pero robusta, para representar el conocimiento de las relaciones causales que afectan un desenlace de interés [19]. Los mapas son gráficos direccionados (*directed graphs* en inglés) que utilizan flechas para mostrar las ideas de los participantes sobre las conexiones causales entre los factores (nodos) y el desenlace. Los participantes indican la fuerza de los vínculos causales con pesos numéricos asignados a cada flecha [20]. Los mapas describen estructuras de creencias compartidas de los participantes utilizando lenguaje cotidiano. La aplicación posterior de la lógica "fuzzy[1]" utiliza el lenguaje matemático [21] para describir estas estructuras como modelos blandos de sistemas de conceptos [22].

[1] Una traducción frecuente del término *fuzzy* es difuso. Aunque este es un término común en la literatura en español, su significado difiere del que *fuzzy* usualmente tiene en inglés. Por esta razón hemos preferido mantener el término original.

El mapeo cognitivo se describió en la década de 1980, [19] basándose en la teoría de gráficas [23] y desarrollos más recientes de la lógica *fuzzy* [24]. La relativa simplicidad de crear los mapas significa que no se limitan a fuentes de conocimiento sofisticadas. Los investigadores han utilizado el mapeo cognitivo para facilitar la participación de las partes interesadas en la planificación ambiental [25-27]. Giles y colegas fueron pioneros en el uso del mapeo cognitivo como una herramienta intercultural para facilitar la comunicación entre la evidencia publicada y el conocimiento indígena, [28] y Dion formalizó este enfoque en el *Peso de la Evidencia* (Weight of Evidence) para contextualizar la mejor evidencia disponible de acuerdo con el conocimiento de las partes interesadas. [29]

Los resultados de las discusiones de nuestros grupos focales sobre *kunika* indicaron que había muchas razones por las que las personas podrían continuar practicando *kunika* a pesar de que sabían que tenía consecuencias adversas. Elegimos utilizar el mapeo cognitivo para explorar con más detalle estas causas de *kunika* por varias razones. Entre otras, porque permite que se construyan sistemas complejos identificando una relación (causa-efecto) a la vez; esto facilita la discusión de sistemas complejos incluso a pesar de diferencias lingüísticas y culturales. El análisis de mapas cognitivos indica la fuerza relativa de las causas directas e indirectas del resultado, destacando los factores clave a abordar para la prevención. Los mapas proporcionan una presentación visual de los hallazgos que pueden ayudar a comunicar los resultados, incluso a personas con conocimientos limitados de aritmética y baja alfabetización.

Muestreo y reclutamiento

Facilitamos 26 sesiones de mapeo. Estratificamos las comunidades en cada uno de los seis distritos en categorías entre urbanos, rurales y rurales remotos, y seleccionamos al azar dos comunidades de cada categoría (12 comunidades en total). Dentro de estas comunidades, los hombres y las mujeres elegibles para participar en los grupos de mapeo eran miembros de la comunidad, habían sido atendidos por el programa de visitas domiciliarias, tenían edad reproductiva o mayor, estaban casados y tenían hijos. En cada comunidad, facilitamos por separado un grupo de mujeres y uno de hombres (24 grupos en total). En algunas comunidades realizamos grupos de hombres y mujeres más jóvenes (menores de 25 años) y en otras realizamos grupos de hombres y mujeres mayores (de 25 años en adelante). Cada grupo de mapeo tenía ocho o nueve miembros. Los miembros del equipo estatal que habían facilitado actividades previas en estas comunidades y estaban familiarizados con las costumbres y tradiciones locales visitaron cada comunidad para reunirse con el jefe de la aldea y el consejo y discutir la actividad con ellos. Los líderes comunitarios se pusieron en contacto, cara a cara, con los posibles participantes e identificaron a los que eran elegibles, estaban disponibles y estaban dispuestos a participar.

También reclutamos a un grupo de proveedores de servicios (hombres y mujeres juntos) que habían trabajado con el equipo en el proyecto de visitas domiciliarias para participar en una sesión de mapeo a nivel de LGA. Un grupo de planificadores de salud, trabajando con el equipo en el proyecto de visitas domiciliarias, participó en una sesión de mapeo a nivel estatal.

Creando los mapas

Dos investigadores locales con fluidez en hausa apoyaron cada sesión de mapeo. Las sesiones comunitarias se llevaron a cabo en un lugar neutral, a menudo la escuela, y las sesiones a nivel del LGA y del estado se llevaron a cabo en oficinas gubernamentales. El facilitador guio el proceso y el reportero tomó notas de la discusión y los significados de los elementos incluidos en los mapas. Los investigadores masculinos facilitaron las sesiones masculinas y las investigadoras facilitaron las sesiones femeninas. Cada

grupo produjo dos mapas: uno de causas de *kunika* y otro de causas de *no-kunika* (*ba kunika* en idioma hausa) o factores preventivos de *kunika*. Producimos mapas separados para las causas y la prevención de *kunika* para ayudar a enfocar la discusión en los grupos.

Cada sesión de mapeo siguió el mismo protocolo y tomó aproximadamente tres horas para producir dos mapas. El facilitador escribió el resultado (*kunika* o *ba kunika*) en una tarjeta magnética ubicada en el centro de un tablero. El facilitador invitó a los participantes, por turnos, a mencionar las causas del resultado de acuerdo con su conocimiento, escribiendo cada causa en tarjetas magnéticas y ubicándolas a su vez en el tablero. Cuando se mencionaron causas duplicadas, estas se eliminaron si todos los participantes estaban de acuerdo en que era una repetición. Muchos de los participantes tenían un nivel de alfabetización limitado o nulo, y el facilitador leía en voz alta lo que escribía en cada tarjeta y, con frecuencia, recordaba a los participantes lo que había en cada tarjeta en el tablero. Esto no fue un problema para los participantes analfabetos, ya que están acostumbrados a confiar en su memoria. Cuando ya no se mencionaron conceptos adicionales, los grupos identificaron relaciones entre factores mientras el facilitador dibujaba las flechas entre conceptos (nodos) para indicar estas relaciones. Cuando el grupo identificó todas las relaciones en el mapa, el facilitador les pidió que identificaran cuál era la flecha más fuerte (peso 5) y cuál era la flecha más débil (peso 1). Utilizando estas dos relaciones ponderadas como referencia, el grupo asignó ponderaciones a todas las demás relaciones.

Al final de la sesión, cada grupo revisó su mapa para confirmar que representaba sus puntos de vista. La secuencia de imágenes de la Figura 1 resume el procedimiento. Los grupos tomaron sus decisiones por consenso. Muchos grupos tuvieron discusiones animadas, por ejemplo, sobre el peso de las diferentes relaciones, pero finalmente todos llegaron a un acuerdo sin necesidad de votar.

Análisis de los mapas

Usamos el software gratuito yEd [30] para digitalizar los mapas. Luego, colocamos las listas de nodos en todos los mapas en una tabla y estandarizamos los nombres de variables, para evitar que los nodos con el mismo significado tuvieran una identidad diferente. Por ejemplo, estandarizamos los nodos denominados "no usar planificación familiar", "planificación familiar no utilizado", "no usar anticonceptivos", "no usar condón" todos como "no usar planificación familiar". Las notas de las sesiones de mapeo ayudaron a aclarar el significado de los nodos cuando hubo dudas. Dos autores (IS y AC) agruparon los factores individuales en categorías más amplias utilizando un enfoque inductivo [31] basado en una interpretación compartida. [32] Los trabajadores de campo que facilitaron las sesiones de mapeo y los miembros del equipo local de investigación luego revisaron y ajustaron los nombres estandarizados de los nodos y las categorías más amplios.

Con los nodos de todos los mapas en un formato estándar, usamos *fuzzy transitive closure* en CIETmap, un paquete de código abierto que proporciona una interfaz con el lenguaje estadístico R, para convertir cada mapa en una red de conocimiento [33]. Luego combinamos los mapas por grupos de mujeres, hombres y funcionarios por separado, utilizando el promedio de los pesos en los mapas individuales después del *fuzzy transitive closure*. El *fuzzy transitive closure* identifica todos los caminos posibles a través de los cuales cada factor podría influir en el resultado de interés y calcula la mayor influencia a través de esos caminos. [34] A partir del análisis de *fuzzy transitive closure*, creamos mapas condensados [26] según las categorías protectoras y causales e indicamos las más influyentes para *kunika*. Estos mapas redujeron el número de factores en menos categorías, definidas en un análisis temático inductivo. Para las relaciones entre categorías, calculamos la influencia neta acumulada como la suma de los pesos de las

influencias en los mapas para esa categoría sobre el peso total acumulado máximo en el mapa condensado. La influencia neta acumulada es una puntuación proporcional entre 1 para las relaciones más fuertes y 0 para aquellas relaciones sin ninguna influencia. Creamos tablas de correspondencia de patrones [20] para comparar la influencia neta acumulada de cada categoría y el número de mapas que mencionaban cada categoría entre los grupos de hombres y mujeres.

Alternativa a la ponderación de los participantes

La ponderación de los participantes de cada causa identificada lleva tiempo, a menudo duplicando la duración de la sesión de mapeo. En algunos entornos, esto no es posible debido al tiempo y otras limitaciones. Probamos una estrategia alternativa, independiente del operador. Para esto, eliminamos los pesos de los mapas antes de combinarlos, de manera que la mención de cada vínculo causal en un mapa individual contribuyera al peso en el mapa combinado. Siguiendo el análisis del discurso de acuerdo con el enfoque original de Harris, [35] tratamos cada vínculo entre un factor causal y un resultado como un morfema (una palabra, parte de una palabra o frase irreductible que tiene significado). De esta manera, al contar la frecuencia con la que se mencionó cada enlace en varios mapas, pudimos ponderar su importancia percibida por los autores del mapa.

Comunicando los mapas

Creamos mapas de resumen que muestran solo las tres categorías más fuertes que causan o previenen *kunika*, con los 48 mapas de la comunidad combinados y para los mapas masculinos y femeninos por separado. Etiquetamos en hausa las categorías de estos mapas resumidos y codificamos por colores las categorías para ayudar a las personas analfabetas a revisar los mapas. En un ejercicio de verificación de los participantes (*member checking*), [36] los facilitadores locales volvieron a reunir a los grupos de mujeres y hombres que habían construido los mapas originales y les presentaron los mapas resumidos de hombres, mujeres y de la comunidad en general. Preguntaron a los participantes si los mapas de resumen representaban fielmente las ideas principales en sus mapas originales. Después, los grupos sugirieron cómo discutir con los hogares de una manera aceptable los temas, a menudo muy sensibles, identificados en los mapas. Estamos preparando un informe aparte sobre estas discusiones y su uso para codiseñar un módulo sobre *kunika* que se incluyó en nuestras visitas domiciliarias a mujeres embarazadas y sus parejas.

Resultados

En total, 97 mujeres y 100 hombres participaron en 48 sesiones de mapeo en seis comunidades rurales y seis urbanas. Todos los participantes estaban casados, con edades entre los 18 y los 60 años para las mujeres y entre 22 y 78 años para los hombres. Un tercio de las mujeres y una cuarta parte de los hombres no tenían educación formal; una cuarta parte de las mujeres y la mitad de los hombres tenían al menos educación secundaria. No fue objetivo específico documentar las reacciones de los participantes en la elaboración de mapas cognitivos, pero todos los facilitadores notaron la participación activa de los participantes y su satisfacción con sus mapas. Crear los mapas y ver reflejados sus conocimientos de esta manera fue una experiencia de validación para los participantes.

Causas de *kunika*

Entre todos los grupos, identificamos una lista de 101 factores causales únicos para *kunika* y los agrupamos en 15 categorías. El Archivo Adicional 1 incluye una tabla de todos los factores únicos en

todos los mapas y las categorías correspondientes del análisis inductivo. La Figura 2 muestra el mapa de causas de *kunika,* después del *fuzzy transitive closure*, para todos los mapas comunitarios combinados. La Figura 3 muestra los mapas separados de hombres y mujeres en las comunidades, el mapa de los funcionarios del LGA y el mapa de los funcionarios a nivel estatal. Para facilitar su interpretación, los mapas de las Figuras 2 y 3 muestran solo las relaciones de las cuatro causas más importantes. El Archivo Adicional 2 incluye una hoja de cálculo con todas las relaciones en los mapas compuestos. Para todos los mapas combinados, las cuatro categorías de riesgo más influyentes para *kunika* fueron:

(1) Relaciones sexuales frecuentes: además de las "relaciones sexuales frecuentes", esa categoría incluía cosas que conducen a relaciones sexuales frecuentes, como un gran deseo, compartir la cama, la presencia del marido en la casa, el amor y la atracción, el uso de maquillaje o ropas ceñidas, mujeres dulces y hombres guapos. También incluyó el uso de medicamentos para el impulso sexual, la pornografía, el descuido y la impaciencia. Incluyó el contacto sexual durante la lactancia y la pérdida de la práctica tradicional de enviar a la esposa a la casa de sus padres después del nacimiento del bebé.

(2) No utilizar métodos modernos de planificación familiar: esta categoría incluía no aceptar métodos modernos de planificación familiar, negarse a recibir inyecciones anticonceptivas, negarse a acudir a los centros de salud o al personal sanitario para recibir asesoramiento sobre anticoncepción y no disponibilidad de productos de planificación familiar.

(3) Dinámica familiar: esta categoría compleja cubría las interacciones entre las parejas y otros miembros de la familia y las presiones y normas sociales que favorecen el intervalo de nacimiento corto y / o las familias numerosas. Incluía tanto la monogamia, porque toda la carga reproductiva recae en una esposa, como los factores relacionados con la poligamia, incluidos los celos y la competencia entre esposas. Incluía el deseo de tener una familia numerosa y el deseo de tener un hijo de un sexo particular. Abarcaba a las mujeres que querían completar su familia rápidamente, la presión social y familiar para tener una familia numerosa y las mujeres que querían satisfacer a sus maridos para que permanecieran fieles.

(4) No usar métodos de planificación familiar no invasivos: Esto incluyó no usar condones, ni el método de *coitus interruptus*, ni el método del calendario, ni los métodos o remedios tradicionales. Algunos grupos enfatizaron que los hombres se negaron a usar estos métodos.

La tabla de patrones (Cuadro 1) destaca las diferencias en las perspectivas de los grupos sobre las causas de *kunika*. Las mujeres y los hombres coincidieron en las tres primeras categorías más influyentes en todos los mapas, según la influencia neta acumulada, aunque el orden de las tres categorías difirió. Para los grupos de mujeres, la dinámica familiar fue la categoría más influyente, seguida del sexo frecuente. La cuarta categoría más influyente identificada por las mujeres fue el uso de la fuerza o la coerción: esto incluía a los hombres que obligaban a las mujeres a tener relaciones sexuales, la dominación masculina y las mujeres que obligaban a los hombres a tener relaciones sexuales.

Los mapas de hombres identificaron el sexo frecuente como la categoría de mayor influencia, seguida de la dinámica familiar. El mapa masculino tenía en la cuarta posición falta de conciencia sobre la planificación familiar y miedo a los efectos secundarios: esto incluía declaraciones sobre el miedo a las complicaciones de los métodos anticonceptivos y el miedo a las complicaciones específicas (como los anticonceptivos que hacen infértiles a las mujeres).

La falta de participación masculina apareció en 11 de los 12 mapas de mujeres, pero en ninguno de los mapas de hombres. De manera similar, el uso de la fuerza o la coacción se mencionó en 11 mapas de mujeres, pero solo en tres mapas de hombres. Por otro lado, la falta de conocimiento sobre *kunika* rara vez aparecía en los mapas de mujeres (un solo mapa) pero era más frecuente en los mapas de hombres (7 mapas). La preocupación por el papel de las condiciones socioeconómicas negativas como causa de *kunika* también fue menos frecuente entre las mujeres (2 mapas) que entre los hombres (9 mapas). Existieron diferencias entre los proveedores de salud a nivel de LGA y hombres y mujeres a nivel comunitario; los proveedores de servicios enfatizaron el menor uso de los servicios de salud, la falta de uso y falta de conocimiento de los métodos de planificación familiar y la falta de participación masculina. Solo el mapa estatal incluyó condiciones socioeconómicas negativas entre las cuatro categorías más importantes de causas de *kunika*.

Causas de no-kunika (factores protectores)

En el mapa compuesto de factores protectores, encontramos 98 factores protectores únicos y los agrupamos en 18 categorías. El Archivo Adicional 2 presenta todos los factores únicos en todos los mapas y las categorías correspondientes del análisis inductivo. La Figura 4 muestra el mapa de factores protectores para *kunika* que surgen del *transitive closure*, para todos los mapas comunitarios combinados. La Figura 5 muestra los mapas separados para hombres, mujeres, funcionarios del LGA y oficiales a nivel estatal. Para facilitar su interpretación, los mapas de las Figuras 4 y 5 muestran solo las relaciones de las cuatro causas más importantes. El Archivo Adicional 3 muestra la lista de todas las relaciones identificadas en los mapas de factores protectores.

Los cuatro principales factores protectores en el mapa general (Cuadro 2) reflejan el inverso de los principales factores causales de *kunika* (Cuadro 1)

(1) Reducir las relaciones sexuales frecuentes: esta categoría incluía ideas como mantenerse alejado del marido durante la lactancia y consejos para evitar las relaciones sexuales durante 4-5 meses después del parto.

(2) Uso de métodos modernos de planificación familiar: esto incluyó el uso de varios métodos modernos de planificación familiar y el asesoramiento al respecto de los trabajadores de la salud. También incluyó el aumento de la demanda de planificación familiar (por ejemplo, mediante el asesoramiento de los trabajadores de la salud), el aumento de la disponibilidad de productos básicos de planificación familiar, la distribución gratuita de productos básicos y el desarrollo de productos básicos con menos efectos secundarios.

(3) Uso de métodos de planificación familiar no invasivos: esto incluyó el uso de condones, método de *coitus interruptus*, método de calendario y métodos tradicionales.

(4) Dinámica familiar: la poligamia fue un factor protector, ya que la carga de tener hijos se comparte entre las co-esposas. Esta categoría también incluyó encontrar formas de evitar la competencia entre co-esposas y reducir la demanda de familias numerosas.

Como se muestra en el Cuadro 2, hubo diferencias entre hombres y mujeres. Hombres y mujeres coincidieron en la importancia de reducir las relaciones sexuales frecuentes y utilizar métodos modernos de planificación familiar. Los mapas de mujeres también destacaron el uso de métodos no invasivos de planificación familiar y la dinámica familiar cambiante, mientras que los mapas de hombres, así como los

mapas de proveedores de servicios y tomadores de decisiones a nivel estatal, enfatizaron la creación de conciencia pública sobre la planificación familiar.

Alternativa a la ponderación de los participantes

La ponderación independiente del observador basada en la aproximación de Harris al análisis del discurso demostró ser un sustituto eficaz de la ponderación de los participantes. Usar la existencia del vínculo (sí / no) en cada uno de los 12 mapas de cada grupo y el conteo de la frecuencia de la ocurrencia produjo esencialmente los mismos resultados que la ponderación de los participantes (consulte el archivo adicional 4).

Comprobación de mapas resumidos por los autores (member checking)

La Figura 6 muestra los mapas de resumen que creamos para la discusión en las comunidades. Los grupos comunitarios de mujeres y hombres que construyeron los mapas originales encontraron que los mapas resumidos se podían relacionar. Los participantes pudieron explicar qué representaban los mapas. Ninguno de los grupos estuvo en desacuerdo con las categorías que combinan grupos de causas y todos los grupos consideraron que los mapas eran una representación justa de su conocimiento sobre las causas de *kunika*.

Cuadro 1. Tabla de patrones de las causas de *kunika*

	Mapa compuesto		Mapas mujeres		Mapas hombres		Mapas LGA		Mapas estatales	
Categorías de causas de *kunika*	INA	n=	INA	n=	INA	n=	INA	n=	INA	n=
Total de mapas	-	24	-	12	-	12	-	1	-	1
Sexo frecuente y factores que lo fomentan	0.78 (1)	24	0.74 (2)	12	0.62 (1)	12	0.40	1	0.07	1
Dinámica de la familia	0.59 (2)	24	0.99 (1)	12	0.22 (2)	12	0.15	1	0.44 (1)	1
No utilizar métodos modernos de planificación familiar	0.26 (3)	23	0.40 (3)	12	0.12 (3)	11	0.50 (2)	1	0.07	1
Falta de conocimiento sobre la PF y miedo a los efectos secundarios	0.13 (4)	20	0.15	11	0.09 (4)	9	0.50 (2)	1	0.14 (2)	1
Uso de fuerza o coerción	0.11	14	0.23 (4)	11	0.02	3	0.25	1	0.04	1
No utilizar métodos de planificación familiar no invasivos	0.11	16	0.18	10	0.04	6	0.00	0	0.00	0
Falta de uso de los servicios de salud	0.08	16	0.07	7	0.06	9	0.60 (1)	1	0.00	1
Condiciones socioeconómicas negativas	0.07	11	0.03	2	0.08	9	0.10	1	0.13 (3)	1
Creencia en la fe y el destino	0.07	15	0.08	7	0.05	8	0.15	1	0.06	1
Planificación familiar ineficaz	0.06	7	0.10	5	0.03	2	0.00	0	0.00	0
Falta de conciencia sobre *kunika*	0.06	8	0.03	1	0.06	7	0.25	1	0.07	1
Falta de participación masculina	0.05	11	0.12	11	0.00	0	0.50 (2)	1	0.06	1
Fertilidad, incluida la fertilidad después del parto	0.05	10	0.09	7	0.01	3	0.15	1	0.07	1
Falta de comunicación conyugal sobre planificación familiar	0.04	7	0.02	2	0.03	5	0.20	1	0.07	1
Falta de estigma alrededor de *kunika*	0.01	3	0.00	0	0.02	3	0.35	1	0.07	1

INA: Influencia neta acumulada sobre *kunika*. Los números entre paréntesis indican el rango de las primeras cuatro categorías más influyentes para cada grupo de partes interesadas. n= número de mapas

Cuadro 2. Tabla de patrones de los factores protectores de *kunika*

	Mapa compuesto		Mapas mujeres		Mapas hombres		Mapas LGA		Mapas estatales	
Categorías de causas de *kunika*	INA	n=	INA	n=	INA	n=	INA	n=	INA	n=
Total de mapas	0.00	24	0	12	0	12	0.00	1	0.00	1
Reducir la frecuencia de relaciones sexuales	0.95 (1)	24	1.00 (1)	12	0.79 (1)	12	0.35 (2)	1	0.08	1
Usar métodos modernos de planificación familiar	0.75 (2)	24	0.99 (2)	12	0.49 (2)	12	0.30 (3)	1	0.25 (4)	1
Usar métodos de planificación familiar no invasivos	0.42 (3)	20	0.75 (3)	12	0.13	8	0.05	1	0.00	0
Dinámica de la familia	0.35 (4)	22	0.60 (4)	12	0.13	10	0.00	0	0.05	1
Mejor comunicación conyugal sobre planificación familiar	0.24	8	0.26	7	0.19 (4)	1	0.14	1	0.15	0
Uso de servicios de salud y asesoramiento de los trabajadores de la salud	0.22	8	0.28	2	0.14	6	0.30 (3)	1	0.28 (3)	1
Crear conciencia pública sobre la planificación familiar y *kunika*	0.20	20	0.05	11	0.27 (3)	9	0.76 (1)	1	0.32 (2)	1
Mayor participación masculina	0.17	11	0.26	2	0.09	9	0.14	0	0.07	1
Aumentar la conciencia sobre *kunika*	0.16	14	0.16	7	0.13	7	0.14	1	0.08	1
Aumentar la conciencia sobre la PF	0.15	16	0.07	9	0.19	7	0.00	1	0.33 (1)	1
Planificación familiar eficaz	0.14	22	0.18	11	0.09	11	0.00	1	0.00	1
Condiciones socioeconómicas	0.11	10	0.13	7	0.08	3	0.11	0	0.17	0
Prevención del sexo forzado	0.10	1	0.21	1	0.01	0	0.03	0	0.00	0
Reducir la fertilidad después del nacimiento	0.04	2	0.09	2	0.00	0	0.11	0	0.02	1
Formas de aumentar la conciencia sobre la PF	0.03	5	0.07	5	0.00	0	0.00	1	0.13	1
Aumentar el estigma sobre *kunika*	0.02	7	0.00	3	0.03	4	0.00	1	0.00	1
Busque el consejo de parteras tradicionales	0.01	1	0.03	1	0.00	0	0.00	0	0.00	0
Creencia en la fe y el destino	0.00	3	0.01	0	0.00	3	0.00	0	0.00	0

INA: Influencia neta acumulada en *no-kunika*. Los números entre paréntesis indican la clasificación de las primeras cuatro categorías más influyentes para cada grupo interesado.

Discusión

Los hombres y las mujeres de las comunidades de Bauchi identificaron fácilmente una serie de factores que consideraron causales y preventivos de *kunika* e indicaron sus puntos de vista sobre la fuerza de los vínculos entre estos factores. Identificaron las causas más influyentes de *kunika* como el contacto sexual frecuente, la dinámica familiar y la falta de uso de métodos anticonceptivos tanto modernos como tradicionales. En mapas separados de factores que protegen contra *kunika*, identificaron como más influyentes las imágenes espejo de estas causas.

Las mujeres y los hombres tenían creencias diferentes. Las mujeres reconocieron el sexo forzado o bajo coacción como una causa importante de *kunika*. Casi todos los mapas de mujeres, pero ninguno de los hombres, mencionaron la falta de participación masculina en la salud reproductiva como una causa de *kunika*. Por otro lado, los mapas de los hombres y los de los proveedores de salud, a nivel estatal y de las LGA, pusieron énfasis en aumentar la conciencia sobre la *kunika* y los métodos de planificación familiar como una forma de reducir la *kunika*.

Las causas de *kunika* identificadas por mujeres y hombres en las comunidades Bauchi difieren de las que se informa que están asociadas con un intervalo intergenésico corto en estudios cuantitativos en países de ingresos bajos y medios. En una revisión sistemática reciente [11], sólo dos factores se asociaron sistemáticamente con un intervalo intergenésico corto en estudios cuantitativos: una duración más corta de la lactancia materna y el sexo femenino del parto anterior. Los factores examinados con más frecuencia en los estudios revisados incluyeron la edad de la madre, la educación de la madre, la educación del padre y la situación socioeconómica del hogar. Algunos de los mapas de nuestro estudio mencionaron el estatus socioeconómico como un factor, pero no como un factor de gran influencia. La edad de la madre y la educación de la madre no figuraban en los mapas, tal vez porque en el contexto de Bauchi la edad temprana de las madres y la baja educación de las mujeres son la norma y no explican por qué algunas mujeres tienen *kunika* y otras no.

Todos los grupos identificaron el sexo frecuente, con factores que conducen a esto, como una causa clave de *kunika*. Algunos mapas identificaron específicamente la reanudación temprana de las relaciones sexuales durante la lactancia como una causa de *kunika*. Los estudios cualitativos, en Nigeria y en otros lugares, han explorado la dinámica del contacto sexual después del nacimiento de un niño y durante el período de lactancia; algunos han señalado el abandono de las prácticas tradicionales y los tabúes durante este período como una causa del intervalo intergenésico corto [37-39]. Algunas de las dinámicas en torno a las relaciones sexuales entre parejas podrían haber cambiado con la transición cultural hacia estilos de vida más modernos y los consiguientes cambios en el comportamiento sexual conservador y el tabú social [37, 38]. Un estudio en el sur de Nigeria encontró que la pérdida de las prácticas de salud tradicionales se asoció con una educación más occidental [40]. Sin embargo, la mayoría de los estudios que examinan la educación materna y el intervalo intergenésico han encontrado un intervalo intergenésico más corto asociado con una menor educación materna [11]. Los grupos de mapeo en Bauchi hablaron abiertamente sobre el uso de afrodisíacos y pornografía como causas de sexo frecuente y, por lo tanto, *kunika*. Se informa que el uso de afrodisíacos a base de hierbas es común entre las mujeres del norte de Nigeria [41, 42]. No tenemos conocimiento de ningún otro estudio que informe que hombres y mujeres relacionen el uso de afrodisíacos con *kunika*.

Los participantes identificaron el uso de anticonceptivos como influyente en la reducción de *kunika*, contrarrestando la influencia del sexo frecuente. La mayoría de los estudios que informan de una asociación entre el uso de anticonceptivos y el intervalo intergenésico en países de ingresos bajos y medianos han encontrado un intervalo intergenésico más largo entre las mujeres que utilizan anticonceptivos [11]. Un análisis de varios países de los datos de las encuestas demográficas y de salud encontró un intervalo intergenésico más corto asociado con el uso de anticonceptivos modernos, posiblemente porque las mujeres comenzaron a usar anticonceptivos después de experimentar un intervalo intergenésico corto no deseado [43].

Las notas de las sesiones que discutieron los mapas resumidos en Bauchi indicaron que los participantes sintieron que las personas no estarían dispuestas a reducir el contacto sexual, pero estarían interesadas en usar anticonceptivos para hacer que tales contactos tengan menos probabilidades de conducir a *kunika*. Las discusiones de grupos focales con mujeres en Nigeria sugirieron que ellas calificaban los riesgos del intervalo intergenésico corto como mayores que los riesgos de los métodos anticonceptivos modernos temporales [44].

Todos los grupos de mapeo identificaron la dinámica familiar como una causa importante de *kunika* e identificaron la modificación de estas dinámicas como crucial para abordar la *kunika*. Estas dinámicas ayudan a explicar por qué las personas pueden continuar practicando *kunika*, incluso cuando saben que tiene consecuencias adversas [10]. La categoría incluye la interacción entre cónyuges (incluidas las interacciones entre co-esposas), las motivaciones para tener más hijos y la influencia de la familia extendida y los vecinos. Las decisiones sobre el espaciamiento de los hijos y el tamaño de la familia son complejas y no se reflejan bien en el concepto estrecho de "necesidad insatisfecha de anticoncepción". Es poco probable que se tenga éxito simplemente instando a las mujeres a usar anticonceptivos, sin considerar las dinámicas familiares conflictivas.

Otros estudios en Nigeria han informado de dinámicas familiares que favorecen un intervalo intergenésico corto, como el deseo de tener una familia numerosa, la necesidad de tener niños como fuerza laboral, la influencia de los suegros y la incertidumbre en torno a la supervivencia de los niños [45, 46]. Los niños, como fuente de orgullo masculino y competencia entre co-esposas, pueden favorecer el intervalo intergenésico corto en Kenia [38]. Consideramos el "deseo de tener un hijo de un sexo particular" como parte de la categoría de dinámica familiar. A menudo, aunque no siempre, se trataba de un deseo de tener un hijo varón cuando el anterior era una niña. Estudios anteriores en Etiopía informaron un intervalo intergenésico más corto si el hijo anterior era mujer [47, 48], y esto también se informó en algunos grupos étnicos en Nigeria [46].

El sexo forzado o coaccionado aparece como una causa influyente de *kunika* en los mapas creados por mujeres, pero no en los creados por hombres. Las notas de los grupos de mapeo sugirieron que a menudo las mujeres comenzaban mencionando el "amor" como causa de *kunika*, pero luego discutieron que las mujeres no podían negarse a tener relaciones sexuales con sus maridos y enfrentaban violencia si lo intentaban. Los niveles de violencia contra las mujeres siguen siendo altos en Nigeria, y en Bauchi son más altos que el promedio nacional [18]. Menos de la mitad de las mujeres de Bauchi pueden negarse a tener relaciones sexuales con sus cónyuges [18]. Un estudio reciente de varios condados con base en los datos de las encuestas demográficas y de salud encontró que la violencia emocional, física o sexual de la pareja íntima se asoció con intervalos entre embarazos más cortos y más embarazos no deseados [49].

Aunque no se identifica como la que tiene la mayor influencia en *kunika*, la falta de participación masculina aparece como una causa de *kunika* en 11 de los 12 mapas de causas de *kunika* de mujeres; pero no estaba presente en ninguno de los mapas de los hombres (Cuadro 1). Esta categoría incluía a los hombres que no estaban dispuestos a usar anticonceptivos ni a recibir consejos al respecto y que no permitían que sus esposas los recibieran. En muchos países africanos, los hombres son tradicionalmente los principales responsables de la toma de decisiones sobre cuándo tener relaciones sexuales, si usar o no anticonceptivos [50, 51] y cuántos hijos tener [52], aunque en los últimos años la decisión conjunta sobre anticoncepción y preferencias de fertilidad se ha hecho más común. [53] Por otro lado, los mapas de hombres dieron más importancia al aumento de la conciencia sobre *kunika* y la planificación familiar como una forma de reducir *kunika*. Esto puede sugerir que son conscientes de su falta de conocimiento sobre estos temas y están dispuestos a involucrarse más.

La construcción y el análisis de mapas cognitivos fue parte de nuestra investigación participativa [54] sobre *kunika* en el estado de Bauchi. Nuestra colaboración con miembros de la comunidad, proveedores de servicios y planificadores de salud comenzó por escuchar las opiniones y el conocimiento de los miembros de la comunidad a través de grupos focales [10] y el mapeo cognitivo que se describe aquí. Continuó compartiendo el conocimiento entre las partes interesadas y proponiendo e implementando soluciones. Nuestro trabajo sobre *kunika* fue estimulado por las autoridades de salud del estado de Bauchi, quienes expresaron su preocupación por la falta de espaciamiento de los nacimientos y sugirieron una exploración de cómo la prevención de *kunika* podría incorporarse al programa de visitas domiciliarias. Ahora planean expandir las visitas domiciliarias, incluido el módulo *kunika*, en todo el estado.

La investigación futura que utilice el mapeo cognitivo podría examinar más a fondo el uso de métodos basados en la frecuencia para ponderar las causas identificadas, lo que según nuestros hallazgos es un enfoque prometedor. También sería interesante examinar con más detalle las causas intermedias y las vías comunes por las cuales diferentes factores impactan el resultado de *kunika*.

Limitaciones

El mapeo cognitivo no significa que las causas identificadas sean causas verdaderas en un sentido absoluto, aunque son causas verdaderas en el entendimiento de quienes construyeron los mapas. La preocupación de los modelos blandos, sin embargo, es involucrar a las partes interesadas en la identificación de cómo abordar el tema de interés, en este caso, *kunika*. Para los participantes, estos mapas ofrecen una forma de presentar y reflexionar sobre lo que saben. Para los investigadores, los mapas son fuente de nuevas hipótesis y variables para dar sentido al tema de estudio.

Todos los participantes en los grupos de mapeo estaban casados y tenían hijos y la mayoría de ellos tenía al menos dos hijos, pero tener dos o más hijos no era un requisito de elegibilidad. Los participantes compartieron sus conocimientos sobre las causas de *kunika* basándose no solo en su propia experiencia, sino también en lo que sabían de la experiencia de la familia y otros miembros de la comunidad.

Usamos el *fuzzy transitive closure* para calcular la influencia de las categorías causales en el resultado. Los mapas compuestos presentan redes complejas de interacciones y es difícil considerar todas las relaciones reportadas. El resultado del *transitive closure* puede parecer complejo, incluso para los investigadores, por lo que era importante crear mapas resumidos que los grupos comunitarios pudieran revisar y usar fácilmente. Nuestros mapas resumidos se centraron en las categorías más sólidas y esta simplificación corre el riesgo de perder información.

El mapeo cognitivo recopila mucha información en poco tiempo. En nuestro caso, la creación de un mapa de causas y otro de factores protectores aumentó el tiempo necesario con cada grupo. Tuvimos un descanso para almorzar entre los dos mapas, pero los participantes pudieron estar cansados y menos comprometidos al crear el segundo mapa. Dados los resultados similares utilizando el análisis del discurso de Harris, las futuras sesiones de mapeo podrían omitir el paso de ponderación de los participantes que, aunque informativo, puede llevar tanto tiempo como la construcción del mapa.

No fue factible regresar a las comunidades e involucrar a los autores del mapa en el análisis temático. Los investigadores que definieron las categorías más amplias de causas y factores protectores utilizaron las notas de la sesión de mapeo y el conocimiento de los trabajadores de campo locales para aclarar el significado de los conceptos. Verificaron sus categorías con el equipo de investigación local y los facilitadores del grupo. Las suposiciones e implicaciones de nuestra categorización son explícitas en el archivo adicional 1. Los grupos comunitarios a los que se les presentaron los mapas resumidos acordaron que estos mapas representaban sus puntos de vista justamente.

Conclusiones

El mapeo cognitivo realizado por miembros de la comunidad en el estado de Bauchi confirma que *kunika* es el resultado de una red compleja de factores que interactúan. Estos incluyen dinámicas específicas de la cultura que los extranjeros que promueven la planificación familiar a menudo ignoran. Aunque la promoción de la anticoncepción tiene un papel que desempeñar en el espaciamiento de los partos, es probable que las estrategias que se basan solo en ella sean insuficientes.

Este estudio demuestra cómo las comunidades pueden tener un papel en la reducción de *kunika*. El énfasis no está en conocer las verdaderas causas de *kunika*, sino en ayudar a las comunidades a identificar lo que saben y lo que pueden hacer para abordar el problema. Los resultados del análisis de mapas cognitivos difusos se pueden hacer accesibles a las partes interesadas, lo que permite su participación significativa en la interpretación y el uso de los hallazgos.

Lista de abreviaturas

INA -- Influencia neta acumulada

LGA -- Área local de gobierno

OMS -- Organización Mundial de la Salud

Declaraciones

Aprobación ética y consentimiento para participar: El Ministerio de Salud del estado de Bauchi dio la aprobación ética para el estudio el 19 de junio de 2017 (NREC / 12/05/2013/2017/21). El IRB de la Facultad de Medicina de la Universidad McGill otorgó la aprobación ética el 25 de septiembre de 2017 (A09-B60-17B). Hombres y mujeres en las sesiones de mapeo dieron su consentimiento informado oral para participar en las discusiones.

Consentimiento para la publicación: Los participantes que aparecen en la Figura 1 dieron su consentimiento firmado para que se publicaran sus fotografías.

Disponibilidad de datos y materiales: Los datos que respaldan los hallazgos de este estudio están disponibles mediante una solicitud razonable a los autores. Los datos se utilizaron para el estudio actual con la autorización de las comunidades participantes, por lo tanto, se requerirá su autorización para compartirlos.

Conflicto de intereses: Los autores declaran que no tienen intereses en competencia.

Fondos: El proyecto está financiado por la iniciativa *Innovating for Maternal and Child Health in Africa* (IMCHA), con el apoyo de *Global Affairs Canada*, los Institutos Canadienses de Investigación en Salud (CIHR) y el Centro Internacional de Investigaciones para el Desarrollo (IDRC). Los autores agradecen a la Red de Investigación en Salud de la Población de Quebec (QPHRN) por su contribución al financiamiento de esta publicación. Los órganos de financiación no desempeñaron ningún papel en el diseño del estudio y la recopilación, análisis e interpretación de los datos ni en la redacción del manuscrito.

Contribuciones de los autores: AC, UA, KO e IS diseñaron el estudio. UA, KO, YG, MCB y AC coordinaron las sesiones de mapeo. AIG propuso el estudio y participó en su diseño y en la interpretación de los hallazgos de las sesiones de mapeo. UA e IS digitalizaron los mapas. IS y AC realizaron el análisis temático inicial y UA, KO, YG y MCB coordinaron el ajuste de la clasificación. De acuerdo con las discusiones durante las sesiones de mapeo. IS y AC realizaron el análisis, produjeron los mapas para discutir en las comunidades y redactaron el manuscrito. NA supervisó la aplicación metodológica del mapeo cognitivo desde el diseño del estudio hasta la presentación de informes. Todos los autores contribuyeron al manuscrito y aprobaron la versión enviada.

Agradecimientos: Los autores agradecen a los miembros del equipo de campo que facilitaron las sesiones, registraron los mapas y prepararon los informes de cada sesión. Agradecemos a las mujeres y hombres que dieron su tiempo y compartieron sus puntos de vista en los grupos de mapeo. Este trabajo fue posible gracias al apoyo del gobierno del estado de Bauchi y la Autoridad del Gobierno Local de Toro y los líderes tradicionales y religiosos de las comunidades.

Detalles sobre los autores: 1 CIET-PRAM, Department of Family Medicine, McGill University, Montreal, Canada. 2 Centro de Investigación de Enfermedades Tropicales (CIET), Universidad Autónoma de Guerrero, Acapulco, Mexico. 3 Federation of Muslim Women Association of Nigeria (FOMWAN), Bauchi State, Nigeria. 4 Bauchi State Primary Health Care Development Agency, Bauchi State, Nigeria

Figura 1 Pasos para construir un mapa cognitivo en una sesión de grupo

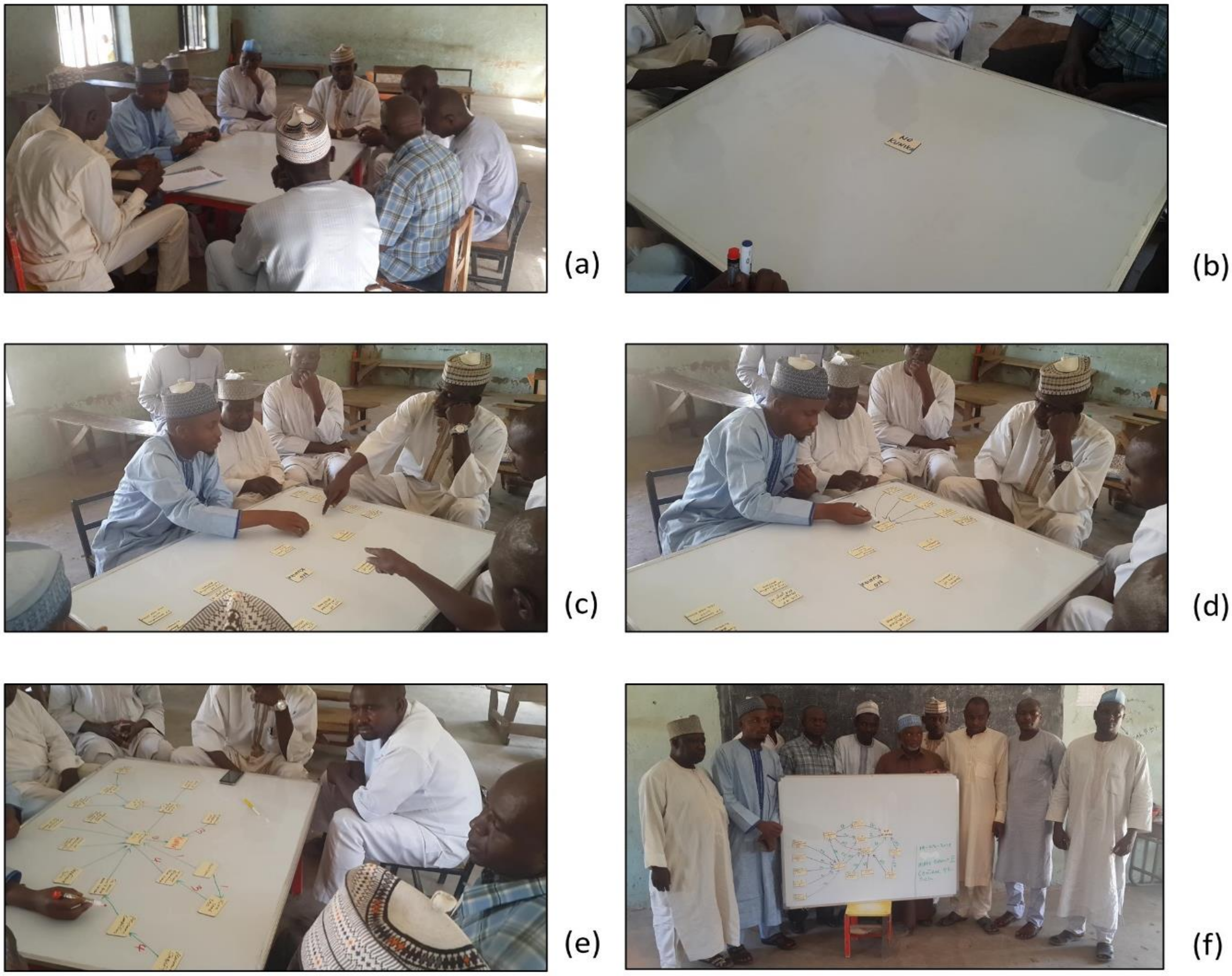

(a) Explicar el proceso al grupo
(b) Identificar el desenlace de interés
(c) Identificar factores (nodos) que causan el desenlace
(d) Dibujar las relaciones (flechas) que unen los nodos
(e) Ponderar la fuerza de cada flecha (1 a 5)
(f) Verificar el mapa con los participantes

Figura 2 Mapa cognitivo compuesto de las causas de *kunika*

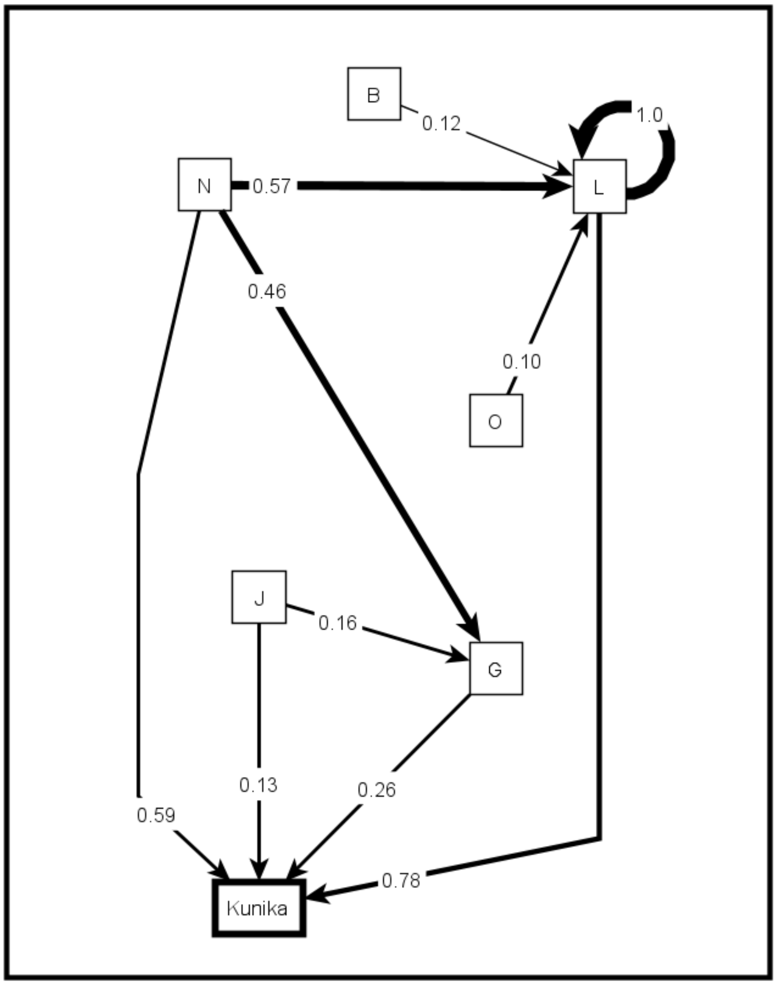

Mapa comunitario compuesto de 24 mapas (12 mapas femeninos y 12 mapas masculinos). Los pesos de las flechas indican la influencia neta acumulada. El grosor de las líneas es proporcional a los pesos. Las flechas que apuntan hacia sí mismas indican que los mapas separados indicaron la dinámica interna de estas categorías, lo que significa que algunos factores dentro de la categoría contribuyen a la ocurrencia de otros factores en la misma categoría. Para simplificar el mapa, incluimos solo las relaciones que afectan las cuatro causas más fuertes de *kunika*. B: Uso de fuerza o coacción; G: No utilizar métodos modernos de planificación familiar; J: Falta de conocimiento sobre la PF y miedo a los efectos secundarios; L: Sexo frecuente y factores que lo fomentan; N: dinámica familiar; O: Condiciones socioeconómicas negativas.

Figura 3 Mapas separados de las causas de *kunika*

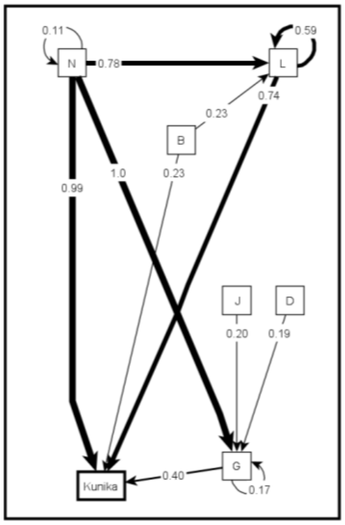

Female Map

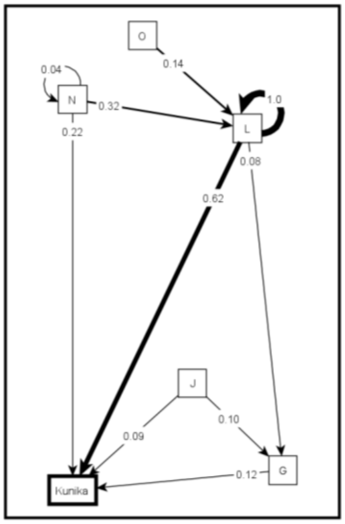

Male Map

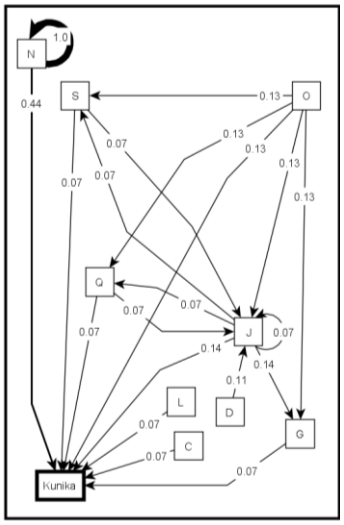

State map

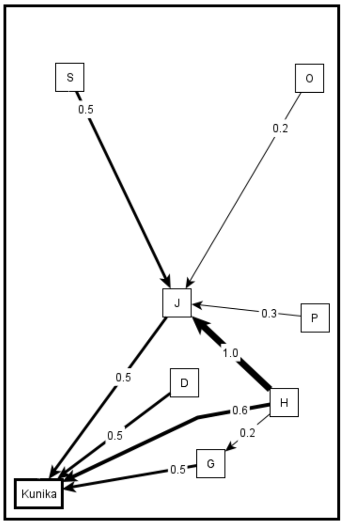

LGA map

El mapa femenino sintetiza 12 grupos femeninos. El mapa masculino sintetiza 12 grupos masculinos. El mapa de estado y el mapa del LGA provienen de una sesión de grupo cada uno. Los pesos de las flechas indican la influencia neta acumulada. El grosor de las líneas es proporcional a los pesos. Las flechas que apuntan hacia sí mismas indican que los mapas separados indicaron la dinámica interna de estas categorías, lo que significa que algunos factores dentro de la categoría contribuyen a la ocurrencia de otros factores en la misma categoría. Para simplificar los mapas, solo incluimos las relaciones que afectan a las cuatro causas más fuertes de *kunika*. B: Uso de fuerza o coacción; C: Falta de comunicación conyugal sobre planificación familiar; D: falta de participación masculina; G: No utilizar métodos modernos de planificación familiar; H: Falta de uso de los servicios de salud; J: Falta de conocimiento sobre la PF y miedo a los efectos secundarios; L: Sexo frecuente y factores que lo fomentan; N: dinámica familiar; O: Condiciones socioeconómicas negativas; P: Creencia en la fe y el destino; P: Falta de conocimiento sobre *kunika*; S: Falta de estigma en torno a *kunika*.

Figura 4. Mapa cognitivo compuesto de los protectores (causas de *no-kunika*)

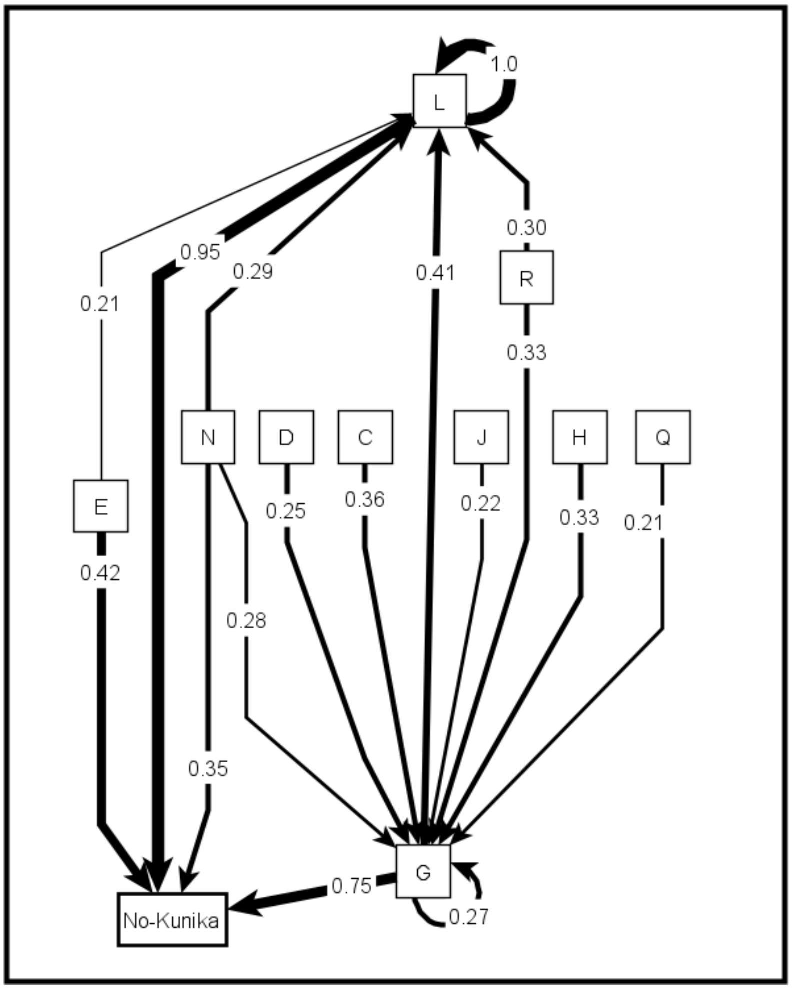

Mapa compuesto de 24 mapas (12 mapas femeninos y 12 mapas masculinos). Los pesos de las flechas indican la influencia neta acumulada. El grosor de las líneas es proporcional a los pesos. Las flechas que apuntan hacia sí mismas indican que los mapas separados indicaron la dinámica interna de estas categorías, lo que significa que algunos factores dentro de la categoría contribuyen a la ocurrencia de otros factores en la misma categoría. Para simplificar el mapa, incluimos solo las relaciones que afectan las cuatro causas más fuertes de *no-kunika*. C: Mejor comunicación conyugal sobre planificación familiar; D: mayor participación masculina; E: Uso de métodos de planificación familiar no invasivos; G: Uso de métodos modernos de planificación familiar; H: uso de servicios de salud y asesoramiento de los trabajadores de la salud; J: Aumentar la conciencia sobre la PF; L: Reducir el sexo frecuente; N: dinámica familiar; P: Incrementar la conciencia sobre *kunika*; R: Crear conciencia pública sobre planificación familiar y *kunika*.

Figura 5. Mapas separados de protectores (causas de *no-kunika*)

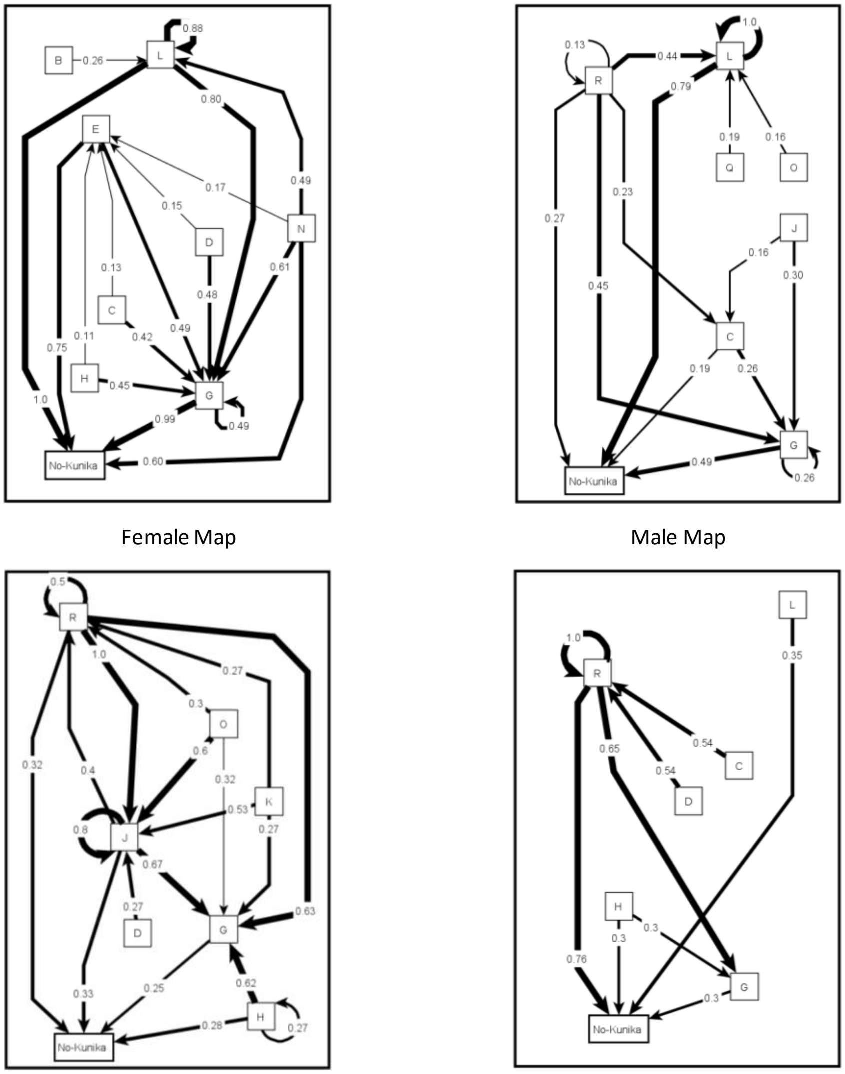

Female Map

Male Map

State map

LGA map

El mapa femenino sintetiza 12 grupos femeninos. El mapa masculino sintetiza 12 grupos masculinos. El mapa de estado y el mapa del LGA provienen de una sesión de grupo cada uno. Los pesos de las flechas indican la influencia neta acumulada. El grosor de las líneas es proporcional a los pesos. Las flechas que apuntan hacia sí mismas indican que los mapas separados indicaron la dinámica interna de estas categorías, lo que significa que algunos factores dentro de la categoría contribuyen a la ocurrencia de otros factores en la misma categoría. Para simplificar los mapas, incluimos solo las relaciones que afectan las cuatro causas más fuertes de *no-kunika*. B: Prevención del sexo forzado; C: Comunicación conyugal sobre planificación familiar; D: Participación masculina; E: Utilizar métodos de planificación familiar no invasivos; G: Utilizar métodos modernos de planificación familiar; H: Uso de los servicios de salud; J: Conocimiento sobre la PF y miedo a los efectos secundarios; K: Formas de aumentar la conciencia sobre la PF; L: Evitar sexo frecuente y factores que lo fomentan; N: dinámica familiar; O: Mejores condiciones socioeconómicas negativas; P: Falta de conocimiento sobre *kunika*; R: Crear conciencia pública sobre planificación familiar y *kunika*.

Figura 6 Mapas resumidos para compartir con grupos de diálogo comunitarios

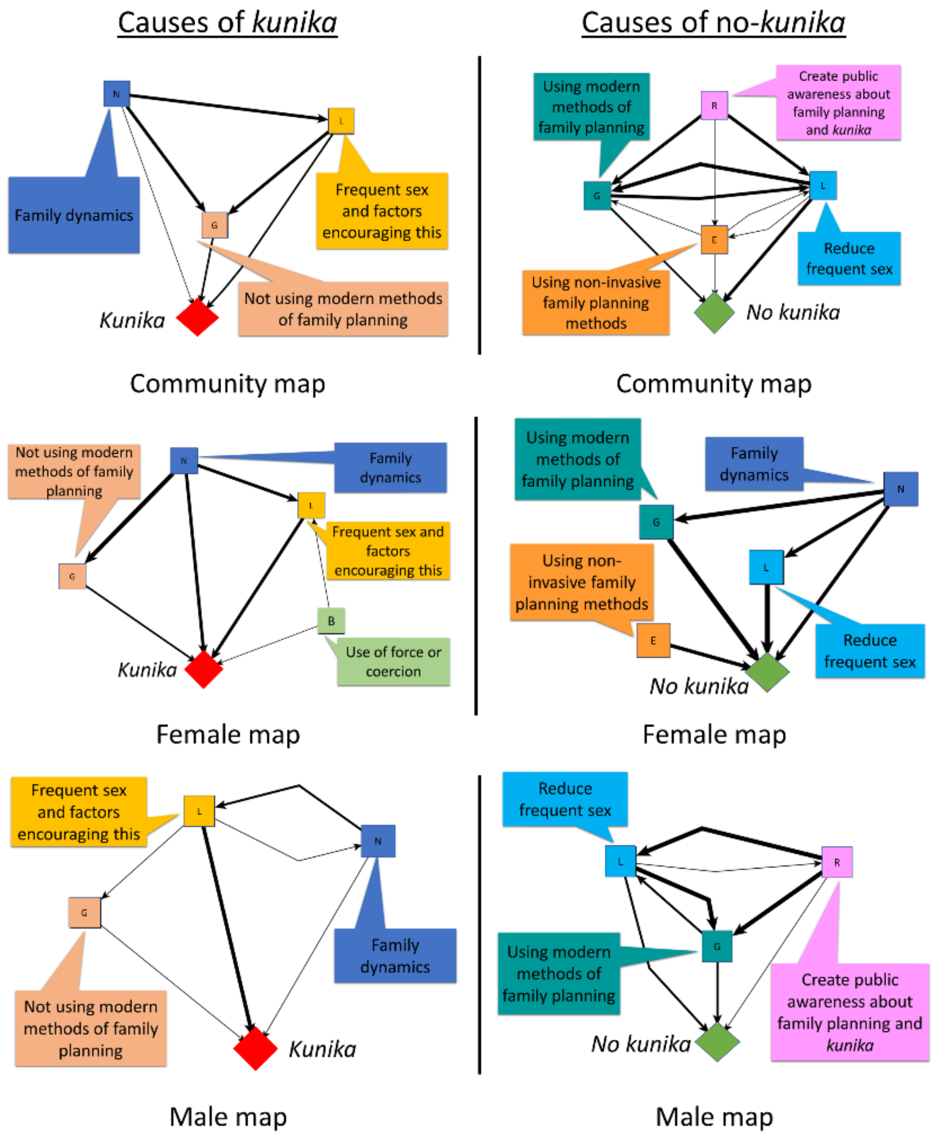

Mapas resumidos para compartir con grupos de diálogo comunitarios. El mapa femenino sintetiza 12 grupos femeninos, el mapa masculino sintetiza 12 grupos masculinos y el mapa comunitario sintetiza todos ellos. El grosor de las líneas es proporcional a la influencia neta acumulada. Para simplificar el gráfico, solo incluimos las relaciones que afectan las tres causas más fuertes de *kunika*. Las etiquetas originales de los mapas estaban en idioma hausa. La codificación de colores ayuda a la interpretación por parte de participantes analfabetos.

Archivos adicionales (disponibles en la página de la versión en inglés del artículo)

Nombre de archivo FCM_Nigeria_Additional_File_1.xlsx

Título de los datos: tabla de factores y categorías coincidentes en todos los mapas

Descripción de los datos: Una tabla que presenta todos los factores con significados similares en la misma fila y la categoría correspondiente asignada durante el análisis temático. Las dos primeras columnas indican los nombres de las categorías y los nombres estandarizados de los factores. Las columnas restantes contienen los factores en el mapa correspondiente. Hay dos hojas, una para causas y otra para protectores.

Nombre de archivo FCM_Nigeria_Additional_File_2.xlsx

Título de los datos: Matrices de adyacencia de mapas compuestos de riesgos por grupo

Descripción de los datos: Cada hoja contiene la matriz de adyacencia del mapa correspondiente. Cada celda de la matriz de adyacencia indica el peso de una relación entre dos categorías. Las filas son la categoría inicial y las columnas la de aterrizaje para cada relación.

Nombre de archivo FCM_Nigeria_Additional_File_3.xlsx

Título de los datos: Matrices de adyacencia de mapas compuestos de factores protectores por grupo

Descripción de los datos: Cada hoja contiene la matriz de adyacencia del mapa correspondiente. Cada celda de la matriz de adyacencia indica el peso de una relación entre dos categorías. Las filas son la categoría inicial y las columnas la de aterrizaje para cada relación.

Nombre de archivo FCM_Nigeria_Additional_File_4.xlsx

Título de los datos: tabla de correspondencia de patrones para comparar mapas ponderados por participantes y mediante análisis del discurso

Descripción de los datos: Existe una tabla para cada mapa compuesto de riesgo y protectores (comunidades, grupos de mujeres y hombres). Cada tabla contiene dos columnas que indican la influencia neta acumulativa de la categoría en *kunika* o *no-kunika* de los mapas originales o del análisis del discurso. Para cada categoría, indicamos el orden relativo a todas las categorías que influyen en el resultado en el mapa.

Referencias

1. Conde-Agudelo A, Rosas-Bermúdez A, Kafury-Goeta AC. Birth spacing and risk of adverse perinatal outcomes. JAMA. 2006;295:1809. doi:10.1001/jama.295.15.1809.

2. Dewey KG, Cohen RJ. Does birth spacing affect maternal or child nutritional status? A systematic literature review. Matern Child Nutr. 2007;3:151–73. doi:10.1111/j.1740-8709.2007.00092.x.

3. Rutstein SO. Effects of preceding birth intervals on neonatal, infant and under-five years mortality and nutritional status in developing countries: evidence from the demographic and health surveys. Int J Gynecol Obstet. 2005;89:S7–24. doi:10.1016/j.ijgo.2004.11.012.

4. World Health Organization. Report of a WHO technical consultation on birth spacing: Geneva, Switzerland 13-15 June 2005. Geneve, Switzerland: World Health Organization; 2007. https://www.who.int/maternal_child_adolescent/documents/birth_spacing05/en/. Accessed 25 May 2020.

5. Molitoris J, Barclay K, Kolk M. When and where birth spacing matters for child survival: an international comparison using the DHS. Demography. 2019;56:1349–70. doi:10.1007/s13524-019-00798-y.

6. Conde-Agudelo A, Rosas-Bermúdez A, Kafury-Goeta AC. Effects of birth spacing on maternal health: a systematic review. Am J Obstet Gynecol. 2007;196:297–308. doi:10.1016/j.ajog.2006.05.055.

7. Conde-Agudelo A, Rosas-Bermudez A, Castaño F, Norton MH. Effects of Birth Spacing on Maternal, Perinatal, Infant, and Child Health: A Systematic Review of Causal Mechanisms. Stud Fam Plann. 2012;43:93–114. doi:10.1111/j.1728-4465.2012.00308.x.

8. Hanley GE, Hutcheon JA, Kinniburgh BA, Lee L. Interpregnancy Interval and Adverse Pregnancy Outcomes. Obstet Gynecol. 2017;129:408–15. doi:10.1097/AOG.0000000000001891.

9. Islamic Perspective on Reproductive Health & Childbirth Spacing in Nigeria. 2017. https://tciurbanhealth.org/wp-content/uploads/2018/12/islamic_perspective_new.pdf.

10. Ansari U, Pimentel J, Omer K, Gidado Y, Baba MC, Andersson N, et al. "*Kunika* women are always sick": views from community focus groups on short birth interval (*kunika*) in Bauchi state, northern Nigeria. BMC Womens Health. 2020;20:113. doi:10.1186/s12905-020-00970-2.

11. Pimentel J, Ansari U, Omer K, Gidado Y, Baba MC, Andersson N, et al. Factors associated with short birth interval in low- and middle-income countries: a systematic review. BMC Pregnancy Childbirth. 2020;20:156. doi:10.1186/s12884-020-2852-z.

12. Duze MC, Mohammed IZ. Male knowledge, attitudes, and family planning practices in Northern Nigeria / Connaissance, attitude et pratiques de la planification familiale chez les hommes au Nigeria du nord. African J Reprod Heal / La Rev Africaine la SantГ© Reprod. 2006;10:53–65.

13. El Hamri N. Approaches to family planning in Muslim communities. J Fam Plan Reprod Heal Care. 2010;36:27–31. doi:10.1783/147118910790291019.

14. Gwarzo TH. Islamic Religious Leaders and Family Planning in Northern Nigeria: A Case Study of Zamfara, Sokoto and Niger States. J Muslim Minor Aff. 2011;31:143–51.

15. Elmusharaf K, Byrne E, O'Donovan D. Social and traditional practices and their implications for family planning: a participatory ethnographic study in Renk, South Sudan. Reprod Health. 2017;14:10. doi:10.1186/s12978-016-0273-2.

16. Cockcroft A, Omer K, Gidado Y, Gamawa AI, Andersson N. Impact of universal home visits on maternal and infant outcomes in Bauchi state, Nigeria: protocol of a cluster randomized controlled trial. BMC Health Serv Res. 2018;18:510. doi:10.1186/s12913-018-3319-z.

17. Cockcroft A, Omer K, Gidado Y, Baba MC, Aziz A, Ansari U, et al. The impact of universal home visits with pregnant women and their spouses on maternal outcomes: a cluster randomised controlled trial in Bauchi State, Nigeria. BMJ Glob Heal. 2019;4:e001172. doi:10.1136/bmjgh-2018-001172.

18. National Population Commission - NPC, ICF. Nigeria Demographic and Health Survey 2018 - Final Report. 2019. http://dhsprogram.com/pubs/pdf/FR359/FR359.pdf.

19. Kosko B. Fuzzy cognitive maps. Int J Man Mach Stud. 1986;24:65–75.

20. Andersson N, Silver H. Fuzzy cognitive mapping: an old tool with new uses in nursing research. 2019.

21. Kosko B. Fuzzy Thinking: The New Science of Fuzzy Logic. New York, NY: Hyperion; 1994. https://books.google.ca/books?id=wIKRGQAACAAJ.

22. Bertalanffy L von. General system theory: foundations, development, applications. Rev. ed. New York: G. Braziller; 1973.

23. Harary F, Norman RZ, Cartwright D. Structural models: an introduction to the theory of directed graphs. New York SE - ix, 415 pages illustrations 24 cm: Wiley; 1965.

24. Zadeh LA. Fuzzy sets. Inf Control. 1965;8:338–53. doi:10.1016/S0019-9958(65)90241-X.

25. Gray S, Gray S, De kok JL, Helfgott AER, O'Dwyer B, Jordan R, et al. Using fuzzy cognitive mapping as a participatory approach to analyze change, preferred states, and perceived resilience of social-ecological systems. Ecol Soc. 2015;20.

26. Özesmi U, Özesmi SL. Ecological models based on people's knowledge: a multi-step fuzzy cognitive mapping approach. Ecol Modell. 2004;176:43–64. doi:10.1016/j.ecolmodel.2003.10.027.

27. Papageorgiou EI, Salmeron JL. A Review of Fuzzy Cognitive Maps Research During the Last Decade. IEEE Trans Fuzzy Syst. 2013;21:66–79. doi:10.1109/TFUZZ.2012.2201727.

28. Giles BG, Findlay CS, Haas G, LaFrance B, Laughing W, Pembleton S. Integrating conventional science and aboriginal perspectives on diabetes using fuzzy cognitive maps. Soc Sci Med. 2007;64:562–76. doi:10.1016/j.socscimed.2006.09.007.

29. Dion A, Carini-Gutierrez A, Jimenez V, Sarmiento I, Ben Ameur A, Joseph L, et al. Weight of Evidence: Incorporating stakeholder knowledge in evidence synthesis to advance mixed methods research. J Mix Methods Res. 2020.

30. yWorks. yED. 2017. https://www.yworks.com/products/yed. Accessed 2 Feb 2018.

31. Thomas DR. A general inductive approach for analyzing qualitative evaluation data. Am J Eval. 2006;27:237–46. doi:10.1177/1098214005283748.

32. Saldaña J. The coding manual for qualitative researchers. Los Angeles, Calif.: Sage Publications Inc.; 2016. https://www.sagepub.com/sites/default/files/upm-binaries/24614_01_Saldana_Ch_01.pdf. Accessed 26 Apr 2019.

33. Andersson N, Mitchell S. Epidemiological geomatics in evaluation of mine risk education in Afghanistan: introducing population weighted raster maps. Int J Health Geogr. 2006;5:1. doi:10.1186/1476-072X-5-1.

34. Niesink P, Poulin K, Šajna M. Computing transitive closure of bipolar weighted digraphs. Discret Appl Math. 2013;161:217–43. doi:10.1016/J.DAM.2012.06.013.

35. Harris ZS. Discourse Analysis. Language (Baltim). 1952;28:1. doi:10.2307/409987.

36. Birt L, Scott S, Cavers D, Campbell C, Walter F. Member Checking: A Tool to Enhance Trustworthiness or Merely a Nod to Validation? Qual Health Res. 2016;26:1802–11.

37. Adeokun LA. Marital Sexual Relationships and Birth Spacing among Two Yoruba Sub-Groups. Africa J Int African Inst. 1982;52:1–14.

38. Dean NR. A community study of child spacing, fertility and contraception in West Pokot District, Kenya. Soc Sci Med. 1994;38:1575–84.

39. Dehne KL. Knowledge of, attitudes towards, and practices relating to child-spacing methods in northern Burkina Faso. J Health Popul Nutr. 2003;21:55–66.

40. Sarmiento I, Zuluaga G, Andersson N. Traditional medicine used in childbirth and for childhood diarrhoea in Nigeria's Cross River State: interviews with traditional practitioners and a statewide cross-sectional study. BMJ Open. 2016;6:e010417. doi:10.1136/bmjopen-2015-010417.

41. Lawal Bello U, Isah J. Use of Herbal Medicines and Aphrodisiac Substances among Women in Kano State, Nigeria. Nurs Heal Sci. 2015;4:41–50.

42. Nigerian "aphrodisiac" potions for a happy marriage. Deutsche Welle. 2019.

43. Ngianga-Bakwin K, Stones RW. Birth intervals and injectable contraception in sub-Saharan Africa. Contraception. 2005;71:353–6.

44. Schwandt HM, Skinner J, Hebert LE, Cobb L, Saad A, Odeku M. Inadequate birth spacing is perceived as riskier than all family planning methods, except sterilization and abortion, in a qualitative study among urban Nigerians. BMC Womens Health. 2017;17:80. doi:10.1186/s12905-017-0439-2.

45. Tsui S, Williamson NE. Child Spacing Attitudes in Northern Nigeria. Part II. Formative Research: Child Spacing and Family Planning Attitudes of Young Married Men and Women in Selected Areas of North West Nigeria. https://pdfs.semanticscholar.org/6020/44623550883efac410ab58c1df81c678f124.pdf. Accessed 25 May 2020.

46. Fayehun OA, Omololu OO, Isiugo-Abanihe UC. Sex of preceding child and birth spacing among Nigerian ethnic groups. Afr J Reprod Health. 2011;15:79–89.

47. Begna Z, Assegid S, Kassahun W, Gerbaba M. Determinants of inter birth interval among married women living in rural pastoral communities of southern Ethiopia: a case control study. BMC Pregnancy Childbirth. 2013;13.

48. Hailu D, Gulte T. Determinants of Short Interbirth Interval among Reproductive Age Mothers in Arba Minch District, Ethiopia. Int J Reprod Med. 2016;2016.

49. Maxwell L, Nandi A, Benedetti A, Devries K, Wagman J, García-Moreno C. Intimate partner violence and pregnancy spacing: results from a meta-analysis of individual participant time-to-event data from 29 low-and-middle-income countries. BMJ Glob Heal. 2018;3:e000304. doi:10.1136/bmjgh-2017-000304.

50. Esber A, Foraker RE, Hemed M, Norris A. Partner approval and intention to use contraception among Zanzibari women presenting for post-abortion care. Contraception. 2014;90:23–8.

51. Mohammed A, Woldeyohannes D, Feleke A, Megabiaw B. Determinants of modern contraceptive utilization among married women of reproductive age group in North Shoa Zone, Amhara Region, Ethiopia. Reprod Health. 2014;11:13. doi:10.1186/1742-4755-11-13.

52. Tilahun T, Coene G, Temmerman M, Degomme O. Spousal discordance on fertility preference and its effect on contraceptive practice among married couples in Jimma zone, Ethiopia. Reprod Health. 2014;11:27. doi:10.1186/1742-4755-11-27.

53. Vouking MZ, Evina CD, Tadenfok CN. Male involvement in family planning decision making in sub-Saharan Africa- what the evidence suggests. Pan Afr Med J. 2014;19. doi:10.11604/pamj.2014.19.349.5090.

54. Andersson N. Participatory research-A modernizing science for primary health care. J Gen Fam Med. 2018;19:154–9. doi:10.1002/jgf2.187.
